# Genome wide analysis of *NLP* transcription factors reveals their role in nitrogen stress tolerance of rice

**DOI:** 10.1038/s41598-020-66338-6

**Published:** 2020-06-10

**Authors:** B. Jagadhesan, Lekshmy Sathee, Hari S. Meena, Shailendra K. Jha, Viswanathan Chinnusamy, Arvind Kumar, Santosh Kumar

**Affiliations:** 1 Division of Plant Physiology, ICAR-Indian Agricultural Research Institute, New Delhi, India; 2 Division of Genetics, ICAR-Indian Agricultural Research Institute, New Delhi, India; 3 IRRI South Asia Regional Centre (ISARC), Varanasi, 221006 Uttar Pradesh India; 4 Division of Crop Research, ICAR Research Complex For Eastern Region, Patna, Bihar India

**Keywords:** Plant sciences, Plant physiology

## Abstract

The NIN-LIKE PROTEIN (NLP) family of transcription factors were identified as nitrate-responsive cis-element (NRE)-binding proteins, which function as transcriptional activators in the nitrate-regulated expression of downstream genes. This study was aimed at genome-wide analysis of *NLP* gene family in rice and the expression profiling of *NLPs* in response to nitrogen (N) supply and deficiency in rice genotypes with contrasting N use efficiency (NUE). Based on *in silico* analysis, 6 *NLP* genes (including alternative splice forms 11 NLPs) were identified from rice. Expression of *NLPs* was promoted by nitrate supply as well as N deficiency (*NLP1, NLP3, NLP4* and *NLP5*). Four rice genotypes APO (high NUE under sufficient N), IR83929-B-B-291-3-1-1 (IR-3-1-1), Nerica-L-42 (NL-42) (High NUE at low N), and Pusa Basmati 1 (PB1, low NUE) to correlate traits governing NUE and expression of *NLPs*. Analysis of rate of nitrate uptake and expression of N assimilatory and uptake genes established that IR-3-1-1 has high uptake and assimilation efficiency, translating into high NUE, whereas PB1 is efficient in uptake only when N availability is high. Along with the transcriptional upregulation of *NLPs*, genotype IR-3-1-1, displayed highest expression of *OsNRT1.1B* gene, the closest rice homologue of nitrate transceptor *AtNRT1.1* and plays major role in nitrate uptake, translocation and signaling in rice. The results showed that high NUE rice genotypes has both high Nitrogen uptake efficiency (NUpE) and Nitrogen utilization efficiency (NUtE), resulting from the effective and coordinated signal transduction network involving the rice homologue of nitrate transceptor *OsNRT1.1B*, the probable primary nitrate response (PNR) regulator *OsNLP1* and the master response regulator *OsNLP3*, a homologue of AtNLP6*/7*.

## Introduction

Nitrogen (N) is an essential nutrient and major component of proteins, chlorophyll, nucleotides and plant hormones, and therefore has immense role in determining plant growth and economic yield^1,2^. In order to meet the food demand of ever-growing human population, enormous amounts of N fertilizers are applied inorder to tap the maximum crop yield potential worldwide^3^. The global demand for N fertilizers in 2014 was 1.13 M tonnes and is projected to grow at approximately 1.4% per year, reaching 1.22 M tonnes by 2020^4^. On the other hand, around 50% of the applied N fertilizer is lost to the environment depending on the cropping conditions and plant species. The loss of fertilizer N results in contamination of soil water and water bodies and production of nitrogenous greenhouse gases like nitrous oxide (N_2_O) which has high global warming potential^5^. Nitrogen use efficiency (NUE) of rice is particularly low (around 40%), though genetic variation for the trait has been reported^6^. Consequently, there is an impending requirement to improve the NUE of rice to maintain the steadiness of high crop yields *vis-a vis* low N fertilizer inputs^7^.

Transgenic manipulation is one of the potent way to achieve the current demand for high NUE, which necessitates comprehensive understanding of mechanisms regulating N uptake, transport, assimilation and signaling^8^. Amongst various N forms that plants are able to take up, nitrate is the most abundant form of N in aerobic agricultural soils and is prone to leaching due to its chemical nature^9^. Recent finding indicates the role of nitrate as a signalling molecule along with its nutritional role in plants^10^. Components mediating nitrate signaling mechanism were recognized in recent past^11^. In particular; the regulator of nitrate assimilation, Nitrilase (NIT2), containing DNA-binding RWP-RK domain, was a found to be a key regulator of nitrate signalling in *Chlamydomonas*^12^. NIT2 protein is structurally similar to NODULE INCEPTION (NIN) proteins of leguminous plants^12^. In higher plants the first NIN gene was identified in *Lotus japonicus* and imperative for symbiotic nodule formation^13^. NIN protein also contains Phox and Bem1 (PB1) domain with the conserved RWP-RK domain, and it is considered the founding member of the NIN-like proteins (NLPs) in plants^14^. Phylogenetic analyses identified homologues of NIN proteins in legumes, NLPs in both legumes and other non- legumes such as rice, Arabidopsis, wheat, and maize^14,15^.

Recently, several studies enhanced the comprehensive understanding on nitrate signalling and its role in the regulation of N uptake and assimilation^16,17^. Konishi and Yanagisawa^18^ deciphered that, AtNLPs play important role in nitrate signaling by bimding to nitrate-responsive cis-elements (NREs) and coordinates nitrate-regulated expression of genes^19^. The discovery of nitrate-CPK-NLP network threw more light on the role of NLPs in nitrate signalling and the importance of phosphorylation of NLPs in nutrient-growth coordination^20^. Liu et al. (2017) also proposed CPK10, CPK30, and CPK32, as major calcium mediated regulators in primary nitrate responses (PNR), and the downstream signaling^20^. Ca^2+^chelator EGTA (ethylene glycol-bis (β-aminoethyl ether)-N, N,N′,N′-tetra acetic acid) could arrest phosphorylation of NLP7 by CPK10. Approximately 50 transcription factor encoding genes including NLP7 were targeted by nitrate-CPK module^21^. These findings clearly points out that manoeuvring Ca^2+^- CPK–NLP signalling cascade is indeed an efficient strategy for improving the NUE^22^. Further, the roles of NLPs in the N starvation adaptation, nodulation, N and phosphate (P) interactions, and root growth have been clarified in recent years^23^. Loss of major part of applied N as gaseous N in the submerged paddies is one of the reason for low NUE in rice^24^. However, aerobic rice soils have a ratio of 6.5:1 of nitrate and ammonical N^25^, indicating a significant role of understanding nitrate signaling for improving rice NUE in drought prone agriculture situations. Current study encompasses the genome wide analysis of *NLPs* from rice, it’s *in silico* expression profiling and expression characterisation in response to N treatments in rice genotypes having contrasting NUE. We hypothesize that differential expression of NLPs may have regulatory role in NUE of contrasting rice genotypes.

## Materials and Methods

### Genome wide identification and Chromosomal localization of NLP homologues

Genome sequence of Rice Genome Annotation Project Release 7 (RGAP)^26^ was Basic local alignment search tool (BLAST) searched using Maize /*Arabidopsis* candidate *NLP* genes. The nucleotide (gene and cDNA) sequences and protein sequences were downloaded for further use. Protein sequences were used for protein BLAST to verify conserved RWP-RK and PB1 domains (RWP-RK. hmm, PF02042; PB1.hmm, PF00564). Information about the physical locations of all identified *NLP* genes on chromosomes was mapped using CIRCOS tool (www.circos.ca/).

### Gene structure prediction and sub cellular protein localisation of NLP homologues

Gene structure of *NLPs*, showing their exon-intron boundaries and UTR regions, was generated using GSDS server (http://gsds.cbi.pku.edu.cn/). Sub-cellular localisation of NLP proteins was predicted using Target P1.1 (www.cbs.dtu.dk/services/TargetP/).

### Identification of NLP homologues and their putative promoter elements

*NLP* sequences were searched in Plant Ensembl Database (www.plants.ensembl.org/) in each rice species and sub-species and then by name/function search. The procedure for identification of homologues and promoter elements were followed as described in Verma et al.^27^. The protein sequences of the identified NLP members were then used as queries in multiple databases to ensure that no additional related genes were missed from the database. Gene and cDNA sequences of 11 rice species, *Oryza barthii, Oryza brachyantha, Oryza glaberrima, Oryza glumipatula, Oryza longistaminata, Oryza meridionalis, Oryza nivara, Oryza punctata, Oryza rufipogon, Oryza sativa Indica Group, Oryza sativa Japonica Group*, were retrieved for further use. Promoter sequence (1000 bp upstream of 5’ UTR) of 11 rice species were retrieved from Plant Ensembl Database (www.plants.ensembl.org/) or Plant PAN (http://plantpan2.itps.ncku.edu.tw) and analysed for the presence of important *cis*-regulatory elements using PLANTCARE (http://bioinformatics.psb. ugent.be/webtools/plantcare/html/).

### Phylogenetic analysis of NLPS from Oryza spp

The protein sequences of putative NLP_S_ downloaded from RGAP and EnsemblPlants (http://plants.ensembl.org/index.html) were used for phylogenetic analysis using Molecular Evolutionary Genetics Analysis software version 7.0 (MEGA7)^28^. For tree construction, the amino acid sequences were aligned using the Clustal Omega (https://www.ebi.ac.uk/Tools/msa/clustalo) program. Neighbour joining analysis was performed with the pair-wise deletion option with Poisson correction, bootstrap analysis was conducted with 1000 replicates and the Poisson correction method was used for computing evolutionary distances.

### *In-silico* expression analysis of NLPS from rice using Genvestigator and NCBI-GEO

Bioinformatics analysis of publically available transcriptome data was used to decipher expression response of *NLPs* in response to various stress treatments and developmental stages. The *in silico* gene expression analysis was done by Genevestigator software^29^ (https://genevestigator.com/gv/index.jsp). The locus IDs of all the genes were given as input in the development and perturbations tool and searched against experiments involving anatomy, development, hormones, nutrients, drought, salt, cold stresses, germination and others. Expression data of relevant experiments (GSE61370 and GSE66807) were downloaded from NCBI-GEO (https://www.ncbi.nlm.nih.gov/gds) and analysed to study *NLPs* expression.

### Analysis of protein-protein interaction networks

To study protein-protein interaction network, NLP protein sequences were analyzed in SMART (http://smart.embl-heidelberg.de/) followed by prediction of interaction partners and networks using STRING tool (http://string-db.org/).

### Plant material

Based on our previous lab and field experiments (unpublished) four rice genotypes APO (high NUE under sufficient N), IR83929-B-B-291-3-1-1 and Nerica-L-42 (High NUE at low N), and Pusa Basmati 1 (PB1) (N responsive and low NUE) were selected for studying the gene expression and N responses. Genotypes were collected from Division of Genetics, ICAR-IARI and Division of Crop Research, ICAR Research Complex for Eastern Region, Patna, Bihar.

### Field evaluation of rice genotypes and determination of NUE

The plants were raised during *Kharif* season (June-Oct 2018) under field conditions with recommended N, 120 Kg ha^−1^ (N+) and without N fertilizer application (N-) at Division of Plant Physiology, ICAR-IARI, New Delhi. Seeds of the rice varieties were washed with double distilled water and then surface sterilized with 0.1% mercuric chloride HgCl_2_ for 5 min. To remove the traces of HgCl_2_, seeds were thoroughly washed for 5-6 times with double distilled water. The surface sterilized seeds were planted in nursery by broadcasting method and 25-30 days old seedlings were transplanted into the field with identification tags for each genotype. The average soil N content before transplanting of crop was 198 Kg ha^−1^ in N + plot and 148 Kg ha^−1^ in N^−^ plot. Full dosage of P_2_O_5_ (as Single Super Phosphate) and K (as Muriate of Potash) was applied as a basal dose. 50% N was applied during field preparation before planting in N + field, remaining 50% was applied as 2 split doses during early- and late- vegetative stages of the crop. At physiological maturity, plants were harvested, oven dried and biomass and grain yield per plant (g) were recorded. Plant material was then powdered and N content of different tissues (leaf, stem and grain) were estimated following Kjeldahl’s method^30^. Nitrogen utilization efficiency (NUtE) was calculated as (g grain/g total N uptake).

### Hydroponic culture rice seedlings for determination of root traits

Plants were raised in hydroponics **(**Supplementary Fig. 1**)** in controlled environment glass house (National Phytotron Facility, ICAR-IARI). Plants were supplied with three different nitrogen treatments, High nitrate: Low ammonia (T1: 6.5 mM Nitrate: 1 mM Ammonium), Low nitrate: High ammonia (T2: 6.5 mM Ammonium: 1 mM Nitrate), Low N (T3: 0.24 mM Ammonium Nitrate). The N treatments were selected based on the previous reports that aerobic rice soils have a ratio of 6.5:1 of nitrate and ammonical nitrogen^31^ and for screening of rice genotypes for low N tolerance, 0.24 mM of N is optimum^32^. Seeds of the rice genotypes were sterilized with 0.1% (HgCl_2_) and wrapped in moistened germination paper. Five days post germination; seedlings having similar growth were used for experimental treatments. The plants were held with cotton plugs in Styrofoam sheets kept on plastic trays containing 10 liters of nutrient solution. Two seedlings were maintained per hill and each treatment comprised of at least three trays. After every 4 days, nutrient solution was changed, pH of the nutrient solution was maintained using a pocket pH meter at 5.0 throughout the study. The growing media was prepared in de-ionized water with modified IRRI nutrient solution (Yoshida et al.,1972) and the composition of the nutrient solution is as follows, 0.035 mM K_2_SO_4_, 0.1 mM magnesium sulphate hepta hydrate (MgSO_4_ 7H_2_O), 0.1 mM calcium chloride (CaCl_2_)_,_ 0.1 mM potassium bi sulphate (KH_2_PO_4_), 0.01 mM micronutrient solution (H_3_BO_3,_ MnCl_2_ 4H_2_O, ZnSO_4_ 7H_2_O, CuSO_4_ 5H_2_O, Na_2_MoO_4_.2H_2_O). Concentration of nitrate and ammonium ions was adjusted depending on the treatments using 1 M potassium nitrate (KNO_3_), 1 M ammonium sulphate ((NH_4_)_2_SO_4_) and 1 M ammonium nitrate (NH_4_ NO_3_). Nitrification inhibitor Dicyandiamide^32^ was also included in the media to prevent conversion of ammonium to nitrate.

After thirty days of transfer to treatment trays, plants were harvested to record different observations **(**Supplementary Fig. 2**)**. For recording root traits, roots were scanned in a root scanner (Epson, Expression 11000XL, Graphic Art Model) representative plants were taken for each replication and scanning was done in triplicates for each treatment. Root scanning data were analyzed by Win-RHIZO, Regent Instruments to calculate total root length, total root volume, total root surface area, average diameter, no of root tips.

### Rate of nitrate uptake in rice genotypes

Seeds of rice genotypes were germinated and transplanted to trays containing nutrient solution as described earlier. Plants were provided with T1 treatment for 2 weeks followed by nitrogen deprivation (0 mM N) for one week. The seedlings were shifted to growth chambers (Model PGW 36, Conviron, Winnipeg, Canada) maintained at: day/night temperature 27 °C/18 °C, photoperiod-10 h, photon flux density of 400 μmol m^−2^ s^−1^ (PAR) and relative humidity (RH) - 80 to 90%. After another week of N deprivation in the growth chambers, seedlings were incubated in 100 µM, and 1.0 mM nitrate concentration for two hours to study kinetics of nitrate uptake. The photon flux density (PAR) was raised to 500 μmol m^−2^ s^−1^ during incubation. Rate of nitrate uptake (µmol NO_3_^−^ g^−1^ FW h^−1^)^33^ was computed based on the nitrate remaining in the incubation solution. Estimation of nitrate in the medium and dried tissue powder was based on hydrazine sulphate reduction method^34,35^. Solution nitrate content was also confirmed using nitrate selective electrode (Go Direct Nitrate Ion-Selective Electrode, Vernier).

### Expression of nitrogen metabolism and NLP genes in rice

Rice genotypes were germinated as described earlier; seedlings were then shifted to glass tubes holding 50 ml nutrient solution and were held with cotton plugs. Two seedlings were maintained per tube and each treatment comprised of at least 25 tubes. The whole experiment was laid out at National Phytotron Facility, Indian Agricultural Research Institute (IARI), New Delhi in growth chambers (Model PGW 36, Conviron, Winnipeg, Canada) maintained at following growth conditions: day/night temperature 27 °C/18 °C, photoperiod-10 h, photon flux density of 400 μmol m^−2^ s^−1^ (PAR) and relative humidity (RH) - 80 to 90%. Plants were provided with T1 treatment for 2 weeks followed by nitrogen deprivation (0 mM N) for two weeks. Plants were then supplied with three different nitrogen treatments, High nitrate: Low ammonia ratio (T1: 6.5 mM Nitrate: 1 mM Ammonium), Low nitrate: High ammonia (T2: 6.5 mM Ammonium: 1 mM Nitrate), Low N (T3: 0.24 mM Ammonium Nitrate) for 24 hours, leaf and root tissues were sampled includes three biological replication in liquid nitrogen for RNA extraction. Since amplicons of NLP1 was not detected in samples after 24-hour exposure to treatments, the time course of *NLP1* expression after 30 min, 2 hr, 24 hr, 48 hr and 72 hr after exposure to 7.5 mM nitrate was analyzed in high NUE genotype IR-3-1-1. Total RNA was extracted using QIAGEN RNeasy plant mini kit followed by DNase I treatment to obtain DNA free RNA. RNA quantification and purity check were done using Thermo nanodrop 2000c spectrophotometer. First strand cDNA was synthesized from 1 µg of total RNA using Superscript–III reverse transcriptase (Invitrogen, USA). To study the expression level of candidate genes, qRT-PCR was carried using Power SYBR Green Master Mix (Applied Biosystems, USA) on real time PCR detection system (Applied Biosystems). qRT-PCR was done using gene specific primers **(**Supplementary Table 1**)**. Melt curve data collection and analysis was enabled. qRT-PCR products were also visualised by agarose gel electrophoresis to confirm the single specific amplicon. Normalization of the data for each transcript was carried out using *OsUBQ1* as an internal control and level of expression were analysed using ∆C_T_ and 2^−∆∆C^T methods^36^.

### Statistical analysis

Two-way analysis of variance (ANOVA) was carried out in GraphPad Prism version 8 (La Jolla, California, USA) with variety, N treatments as treatment effects to compute adjusted P values and level of significance. Mean separation was done using Sidak’s multiple comparisons test following one-way ANOVA^37^ (Supplementary Tables 2–5**)**. Graphs and heatmaps were prepared using GraphPad Prism version 8 (La Jolla, California, USA) and illustrations in Fig. 9 was made with Biorender (https://biorender.com/).

**Figure 1:**
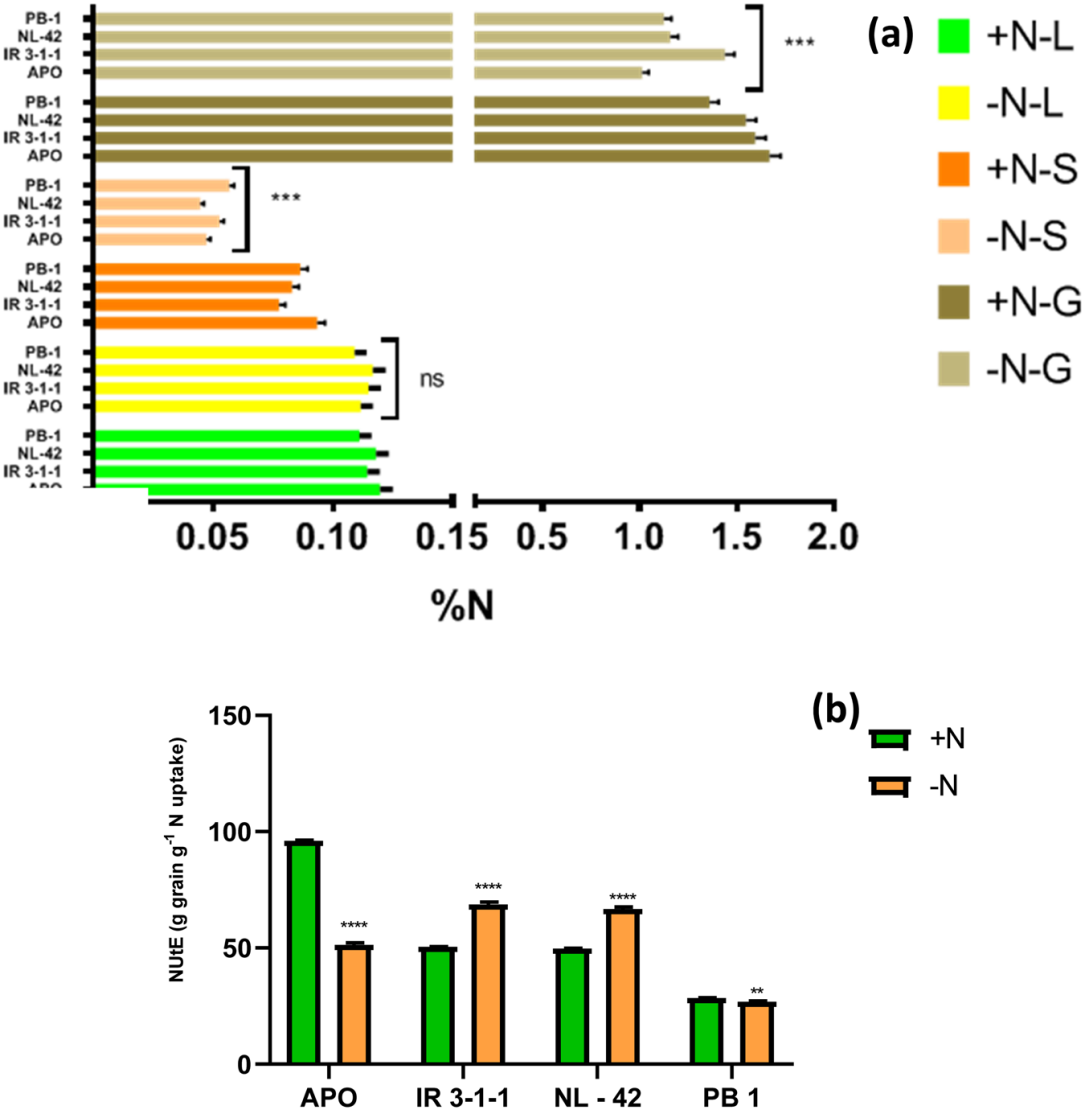
Effect of nitrogen deficient (no applied N: N−) and nitrogen sufficient (120 kg ha^−1^ applied N: N+) field conditions on **(a)** Tissue N% (L-leaf, S-culm, G-grain) **(b)** nitrogen utilization efficiency (NUtE) of rice genotypes Apo, IR-83929-B-B-291-3-1-1 (IR-3-1-1), and Nerica L-42 (NL-42), and Pusa Basmati 1 (PB1). Values are means (±SE) of 3 biological replicates. Sidak’s multiple comparisons test for influence of +N and –N is indicated with astericks. (P values less than 0.001 are summarized with three asterisks, and P values less than 0.0001 are summarized with four asterisks).

**Figure 2:**
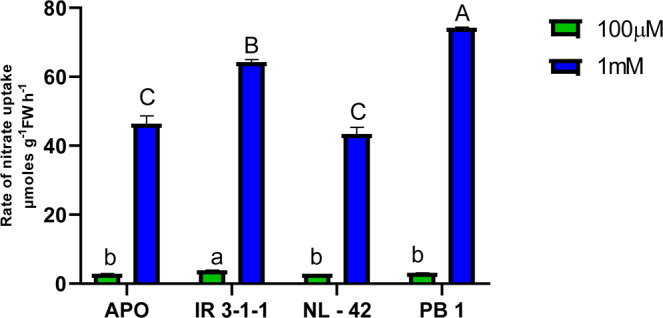
Comparison of rate of nitrate uptake (µmol g^−1^ FW h^−1^) of rice genotypes Apo, IR-83929-B-B-291-3-1-1 (IR-3-1-1), and Nerica L-42 (NL-42), and Pusa Basmati 1 (PB1) in hydroponics and receiving 0.1 and 1.0 mM nitrate treatments. Values are means (±SE) of 3 biological replicates. Duncan’s multiple comparisons test for varietal differences indicated with different letters for each nitrate level.

**Figure 3:**
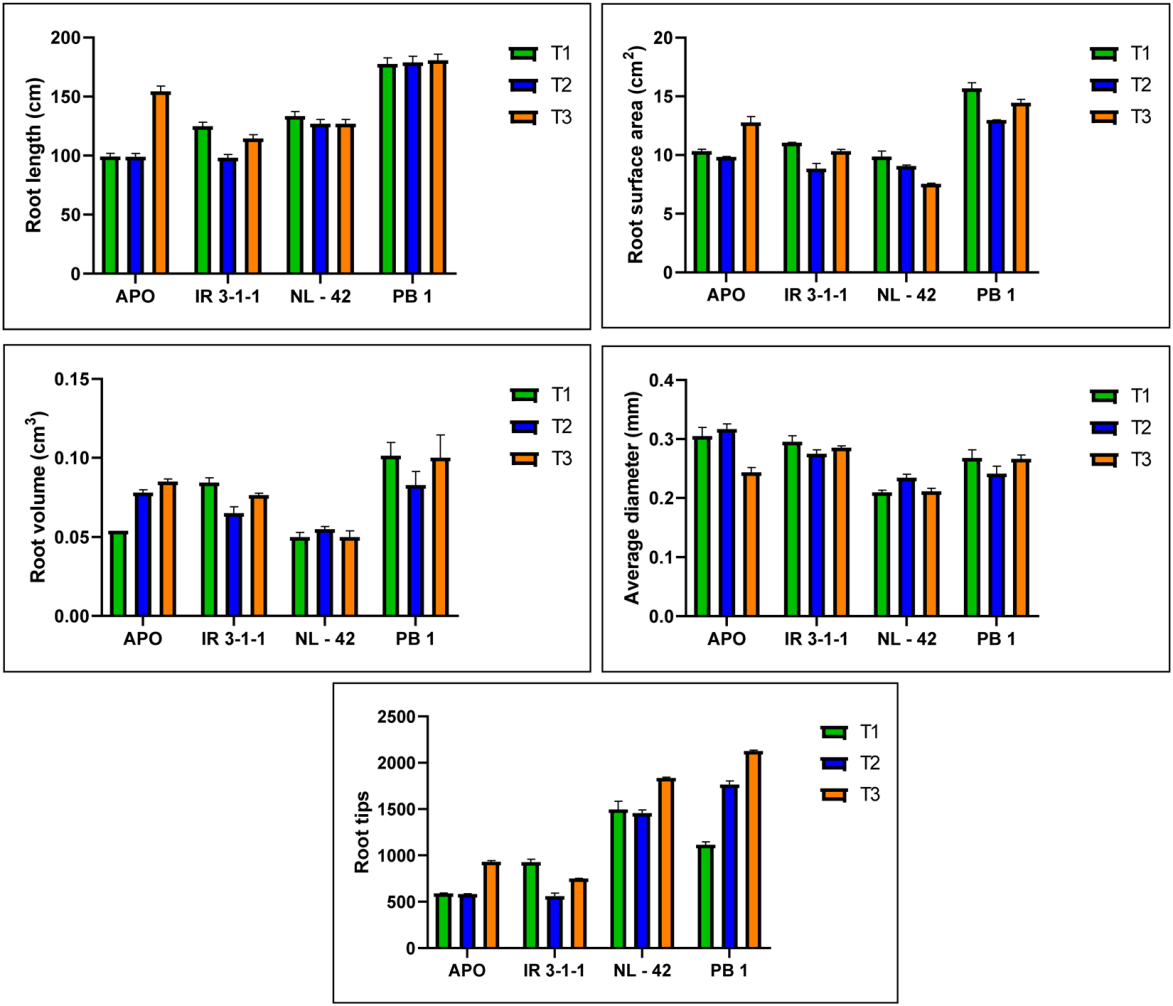
Comparison of total root parameters of rice genotypes Apo, IR-83929-B-B-291-3-1-1 (IR-3-1-1), and Nerica L-42 (NL-42), and Pusa Basmati 1 (PB1) in hydroponics and receiving different nitrogen treatments; T1: 6.5 mM Nitrate: 1 mM Ammonium, T2: 6.5 mM Ammonium: 1 mM Nitrate, T3: 0.24 mM Ammonium Nitrate. Values are means (±SE) of 3 biological replicates. Values are means (±SE) of 3 biological replicates. Sidak’s multiple comparisons test for varietal differences indicated with astericks. (P values less than 0.001 are summarized with three asterisks, and P values less than 0.0001 are summarized with four asterisks).

**Figure 4:**
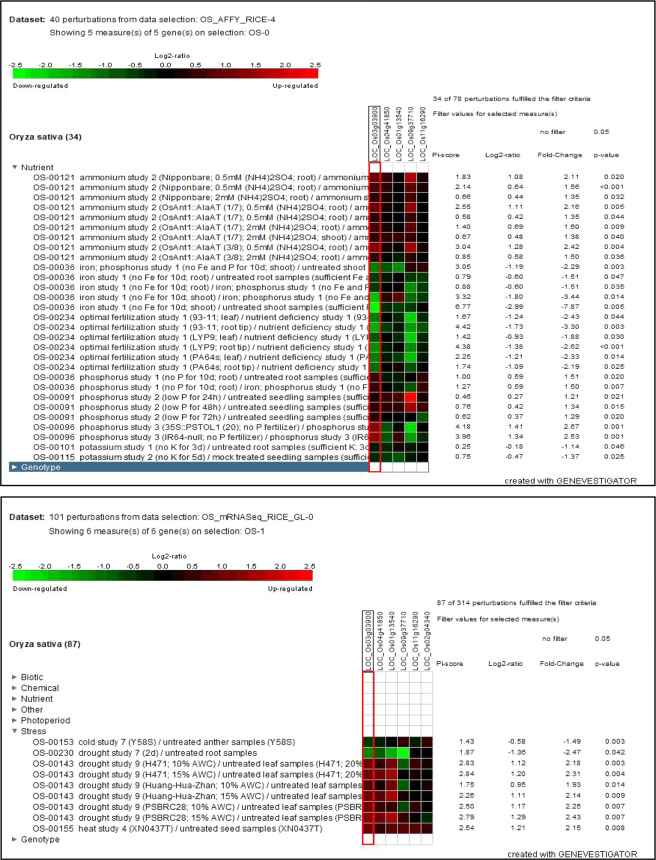
Expression analysis of *NLP* genes in response to nutrient (**a**) and abiotic stress (**b**) perturbations showing significant expression changes at P value ≤ 0.05 (**b**) using Genevestigator database.

**Figure 5:**
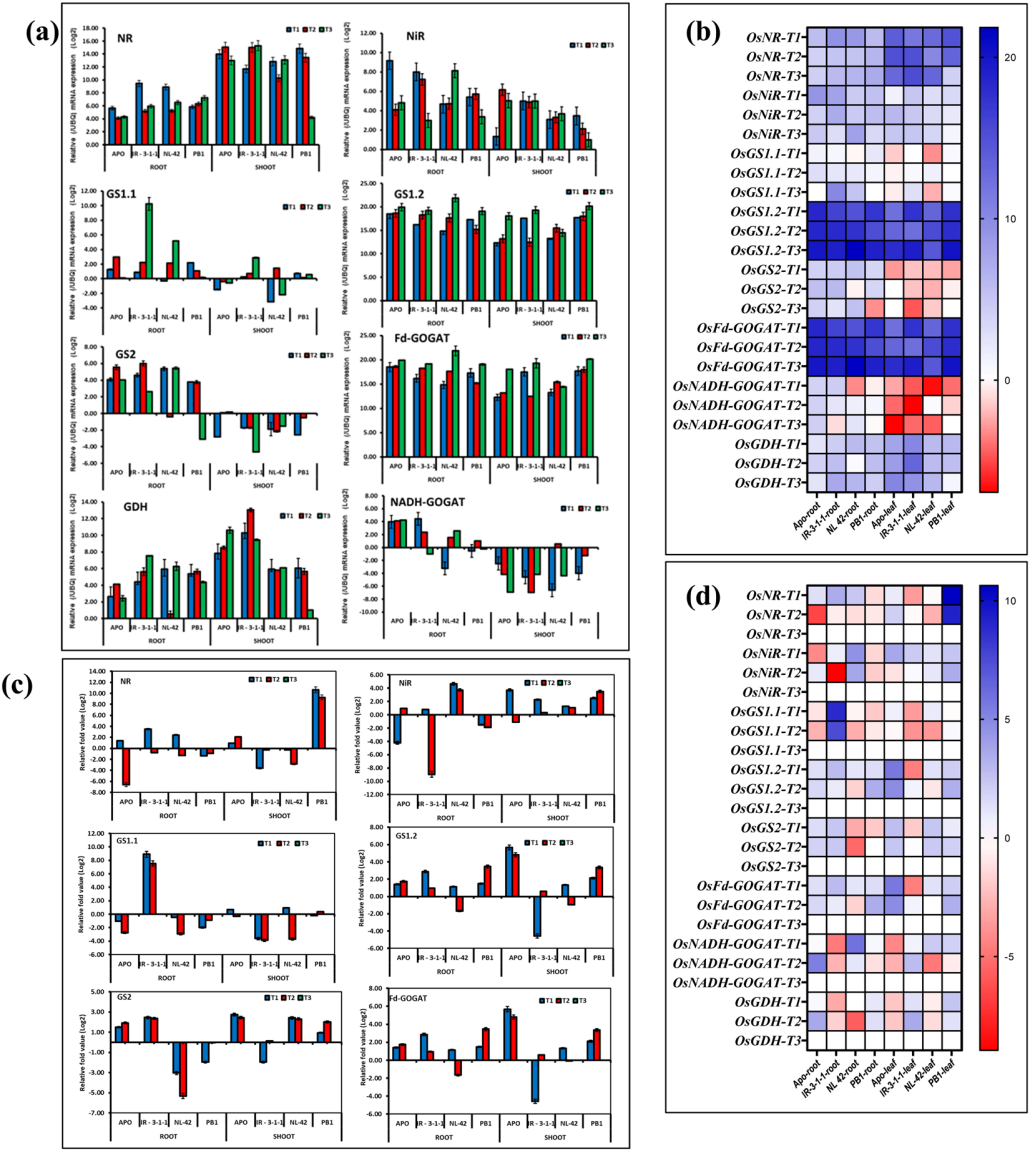
Relative mRNA expression (/UBQ) (**a**,**b**) and relative expression of (w.r.to T3 treatment) (**c**,**d**) of nitrogen assimilation genes in leaves and roots of rice genotypes Apo, IR-83929-B-B-291-3-1-1 (IR-3-1-1), and Nerica L-42 (NL-42), and Pusa Basmati 1 (PB1) in hydroponics and subjected to nitrogen treatments. High nitrate: Low ammonia ratio (T1: 6.5 mM Nitrate: 1 mM Ammonium), Low nitrate: High ammonia (T2: 6.5 mM Ammonium: 1 mM Nitrate), Low N (T3: 0.24 mM Ammonium Nitrate). Values are means (±SE) of 3 biological replicates.

**Figure 6:**
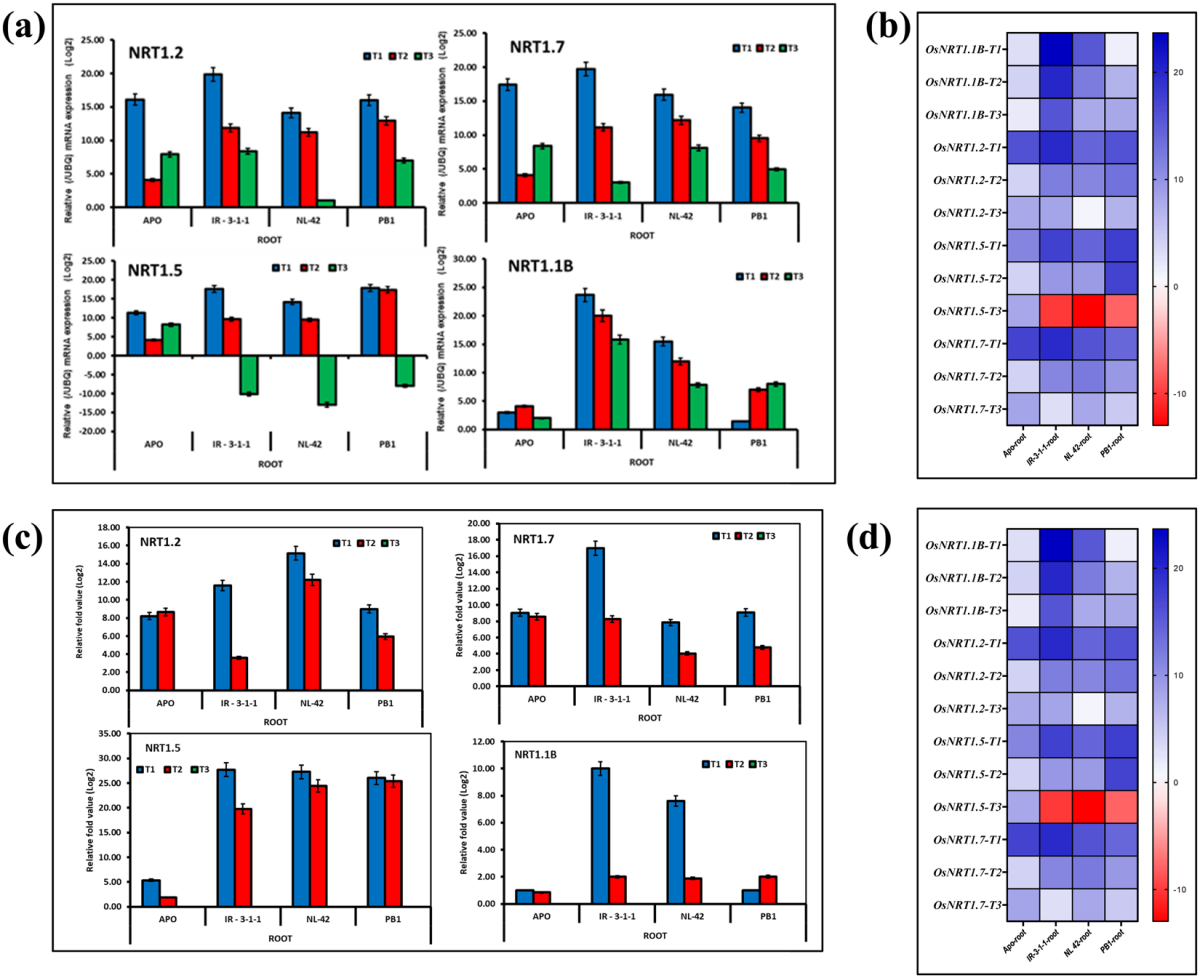
Relative mRNA expression (/UBQ) (**a**,**b**) and relative expression of (w.r.to T3 treatment) (**c**,**d**) of nitrogen uptake genes in leaves and roots of rice genotypes Apo, IR-83929-B-B-291-3-1-1 (IR-3-1-1), and Nerica L-42 (NL-42), and Pusa Basmati 1 (PB1) in hydroponics and subjected to nitrogen treatments. High nitrate: Low ammonia ratio (T1: 6.5 mM Nitrate: 1 mM Ammonium), Low nitrate: High ammonia (T2: 6.5 mM Ammonium: 1 mM Nitrate), Low N (T3: 0.24 mM Ammonium Nitrate). Values are means (±SE) of 3 biological replicates.

**Figure 7:**
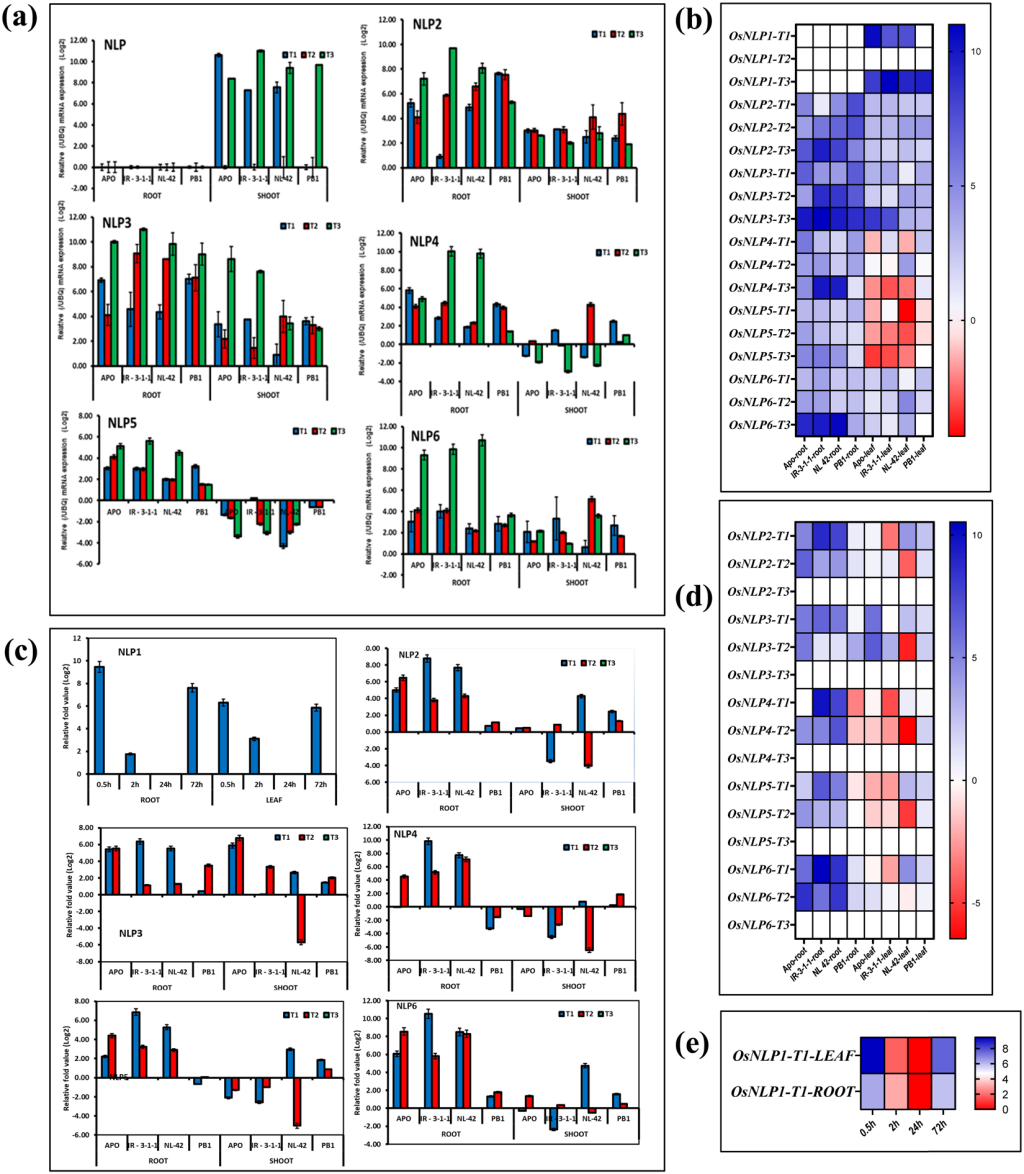
Relative mRNA expression (/UBQ) (**a**,**b**) and relative expression of (w.r.to T3 treatment) (**c**,**d**) *NLP* genes in leaves and roots of rice genotypes Apo, IR-83929-B-B-291-3-1-1 (IR-3-1-1), and Nerica L-42 (NL-42), and Pusa Basmati 1 (PB1) in hydroponics and subjected to nitrogen treatments. High nitrate: Low ammonia ratio (T1: 6.5 mM Nitrate: 1 mM Ammonium), Low nitrate: High ammonia (T2: 6.5 mM Ammonium: 1 mM Nitrate), Low N (T3: 0.24 mM Ammonium Nitrate) and (**e**) time course of *NLP1* relative expression in roots of IR-3-1-1 exposed to T1 treatment.Values are means (±SE) of 3 biological replicates.

**Figure 8:**
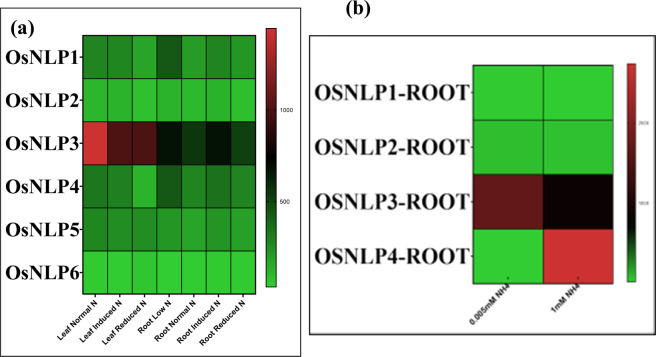
Expression heat map of (**a**) experiment GSE61370 (rice roots exposed to 0.005 and 1 mM NH_4_Cl for 10 days) (**b**) GSE66807 (low N adapted rice plants (0.3 mM NO_3_) transferred to normal (high, 3 mM NO_3_) N condition (induction treatment) and plants grown with normal N conditions moved to a low N system (reduction). Retrieved from publically open database (https://www.ncbi.nlm.nih.gov/gds).

**Figure 9:**
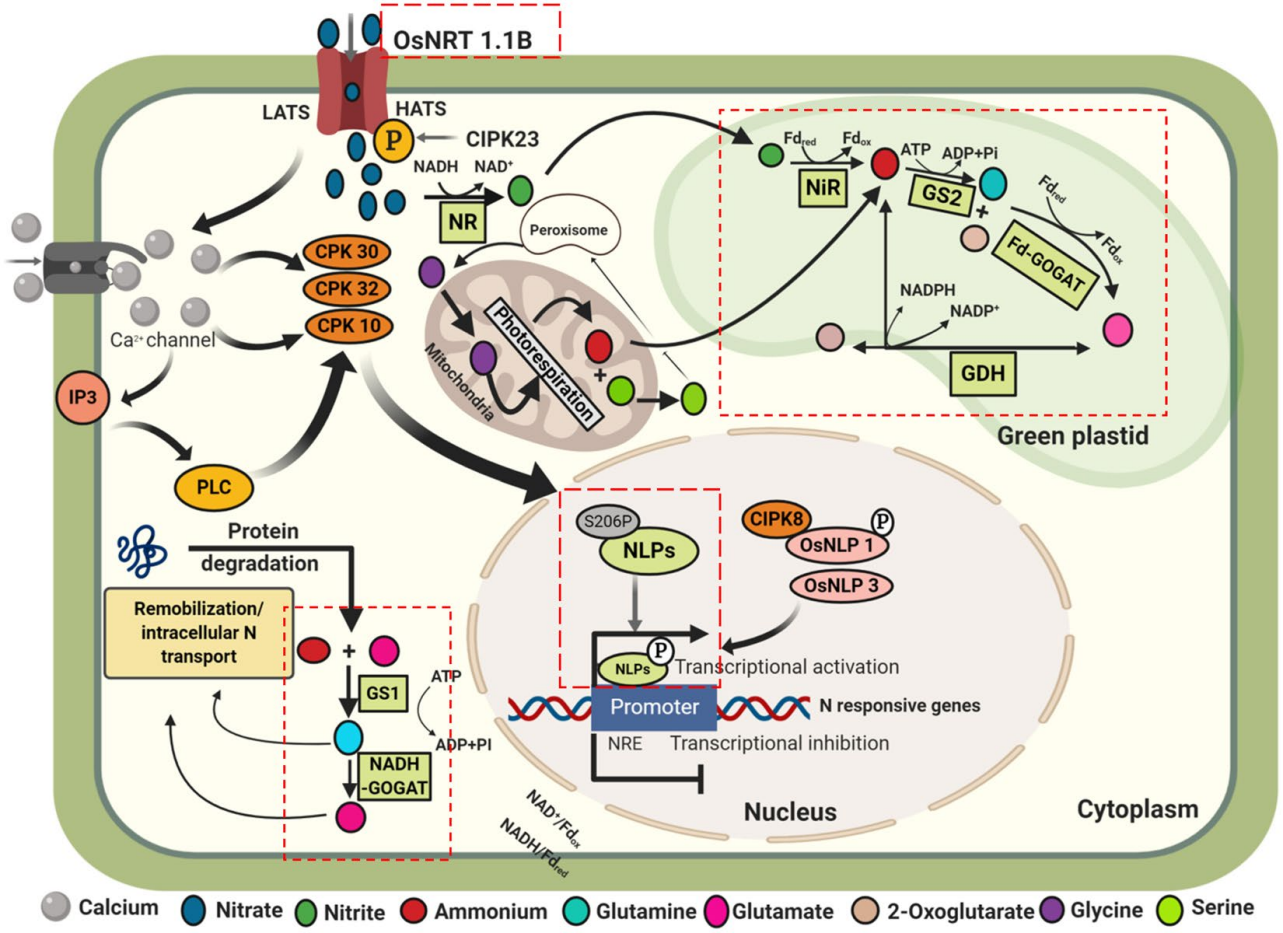
Schematic model summarizing gene regulation by nitrogen in plants. Illustrations was made with Biorender (https://biorender.com/) based on the information available in the public domain. The transcriptional changes of the steps analyzed in the study are enclosed in dashed outlines.

## Results

### Rice genotypes showed variation in different traits governing NUE

To correlate the role of NLPs in governing NUE of rice, we used four rice genotypes identified based on previous lab studies (unpublished). The variability in traits governing NUE such as N content (N%), rate of nitrate uptake, root system architechture and NUE are presented.

Plants grown under N- showed overall decrease in N % of leaves, culm and grain in all the genotypes. However, differences in treatment means were significant only in culm and grains. In culms there was 40% reduction in N content in N- w.r.to N + treatment, whereas in grains the reduction was 23%. There was significant treatment effect on % reduction in varietal means of culm and grain N%: APO (49% and 39%), IR-3-1-1 (32% and 9%), NL-42 (46% and 25%) and PB1 (34% and 17%) as shown in Fig. 1a. Under low N, maximum culm N was shown by PB1 (indicating impaired N remobilization). Highest Grain N% was displayed by IR-3-1-1 followed by NL-42. Varietal means of NUtE **(**Fig. 1b**)** were significantly different as indicated by Sidak’s multiple comparisons test: with respect to N + treatment N- treatment means decreased by 46% and 5.6% in APO and PB1 whereas increased by 36% and 34% in IR-3-1-1 and NL-42 respectively.

The rate of nitrate uptake was studied at 2 h after incubating rice seedlings in either at high (1 mM) or low (100 µM) nitrate concentrations **(**Fig. 2**)**. Significant difference in rate of uptake was observed among genotypes, IR-3-1-1 and APO displayed higher rate of uptake in response to low nitrate supply. Whereas IR-3-1-1 and PB1 were more efficient in taking up nitrate as the nitrate availability was higher. PB1 being a N responsive genotype is efficient in uptake when N availability is high, IR-3-1-1 has high uptake efficiency, at both high and low external nitrate concentrations.

Rate of nitrate uptake is often determined by root system responses. Significant difference was observed in root traits viz., total root length, total root volume and total root surface area with N treatments and varieties **(**Fig. 3a–e**)**. Nitrate nutrition (T1) and n deficiency (T3) triggered root growth and root surface area in rice. N deficiency also significantly promoted root proliferation. Genotypic variation in root traits was also significant. Root proliferation also has significant correlation with nitrate uptake kinetics. Genotypes PB1 and IR-3-1-1 were having statistically significant robust root system. The treatment effects on root diameter were not significant; however, the genotypic effects were significant.

### Genome wide identification and analyses of rice NLP homologues

Genome sequence of Rice Genome Annotation Project Release 7 (RGAP^26^) was BLAST searched for *Arabidopsis* candidate *NLP* genes. Protein sequences were used for protein BLAST to verify conserved RWP-RK and PB1 domains (RWP-RK. hmm, PF02042; PB1.hmm, PF00564). Six *NLPs* were identified from *Oryza sativa* Japonica. These genes were named as *NLP1* to *NLP 6* based on similarity to *Arabidopsis NLPs* and previous reports in rice^14^. Including alternative splice forms *11 NLPs* were identified in rice genome **(**Table 1) Gene structure of *NLP* genes, showing their exon-intron boundaries and UTR regions, was generated using GSDS server. Number of exons ranged from 1 in *NLP5.5* to *7* in *NLP6*. There were four exons in *NLP1*, five exons each in *NLP2* and *3*, four in *NLP4* (Supplementary Fig. 3a). Information about the physical locations of all identified *NLP* genes on chromosomes (Chr.) was mapped using CIRCOS tool (www.circos.ca/) (Supplementary Fig. 3b). *NLPs* were located in 6 out of 12 chromosomes in rice: *NLP3* in Chr.1, *NLP6* in Chr.2, *NLP2* in Chr.4, *NLP4* in Chr.9, *NLP5* in Chr.11 and *NLP1* in Chr.3. Subcellular localisation of NLP proteins was studied by using Target P **(**Table 2). Location with the highest score is the most likely according to Target P. Except NLP5.3, NLP5.5 (predicted as a secretory peptide) and NLP5.4 (predicted as mitochondria localised), NLPs are preferably having either cytosolic or nuclear localisation in agreement with *At* NLPs.

**Table 1:** Details of *NLP* homologues in *Oryza sativa* Japonica.

Gene name	Locus ID	Locus ID Of alternative splice forms
*OsNLP1*	LOC_Os03g03900.1	—
*OsNLP2*	LOC_Os04g41850.1	—
*OsNLP3*	LOC_Os01g13540.1	—
*OsNLP4*	LOC_Os09g37710	LOC_Os09g37710.1(NLP4.1) LOC_Os09g37710.2 (NLP4.2)
*OsNLP5*	LOC_Os11g16290	LOC_Os11g16290.1 (NLP5.1) LOC_Os11g16290.2 (NLP5.2) LOC_Os11g16290.3 (NLP5.3) LOC_Os11g16290.4 (NLP5.4) LOC_Os11g16290.5 (NLP5.6)
*OsNLP6*	LOC_Os02g04340.1	—

**Table 2:** Protein localization of NLP homologues in *Oryza sativa* Japonica predicted by TargetP 1.1 Server. Reliability Class (RC) is a measure of the size of the difference (‘diff’) between the highest (winning) and the second highest output scores.

Name	Len	Chloroplast Transit Peptide (cTP)	Mitochondral targeting Peptide mTP	Signal Peptide (SP)	Others	Loc	Reliability Class (RC)
*OsNLP1*	942	0.187	0.084	0.051	0.897	—	2
*OsNLP2*	936	0.087	0.114	0.046	0.920	—	1
*OsNLP3*	938	0.281	0.037	0.012	0.884	—	2
*OsNLP4.1*	842	0.066	0.121	0.040	0.847	—	2
*OsNLP4.2*	877	0.144	0.055	0.031	0.902	_	2
*OsNLP5.1*	886	0.187	0.034	0.086	0.566	_	4
*OsNLP5.2*	858	0.187	0.034	0.086	0.566	_	4
*OsNLP5.3*	329	0.009	0.063	0.801	0.105	S	2
*OsNLP5.4*	299	0.081	0.677	0.011	0.392	M	4
*OsNLP5.5*	274	0.012	0.165	0.854	0.171	S	2
*OsNLP6*	669	0.022	0.284	0.048	0.898	—	2
**Cut-off**		**0.730**	**0.860**	**0.430**	**0.840**		

There are 5 reliability classes, defined as 1: diff > 0.800, 2: 0.800 > diff > 0.600, 3: 0.600 > diff > 0.400, 4: 0.400 > diff > 0.200, 5: 0.200 > diff.

The protein sequences of putative *OsNLP*_*S*_ were downloaded from RGAP, and used for BLAST search of *Arabidopsis thaliana* genome (TAIR10) at Ensembl Plants (http://plants.ensembl.org/index.html) to identify the homologs of *OsNLPs* from *Arabidopsis* and other eleven rice spp. The protein sequences of rice NLPs and their *Arabidopsis* homologs were used for phylogenetic analysis using Molecular Evolutionary Genetics Analysis software version 7.0 (MEGA7)^28^. Based on phylogenetic analysis rice *NLPs* were classified in to 3 groups. Group I is the smallest group consists of four rice (*NLP6, NLP6.1, NLP5, NLP5.1*) and three Arabidopsis *NLPs* (*NLP1, NLP2 and NLP3*). Group II consists of twenty one rice (*NLP1, NLP3, NLP4*) and two Arabidopsis *NLPs* (*NLP4* and *NLP5*). Group III consists of forty-two rice (*NLP2, NLP3, NLP4, NLP5, NLP6*) and four *Arabidopsis* (*NLP6, NLP7, NLP8* and *NLP9*) *NLPs* (Supplementary Fig. 4). Based on the bootstrap values and tree branching, rice *NLP3 and NLP4* and *Arabidopsis NLP6* and *NLP7* can be considered as paralogs.

The protein sequences of the identified NLP members were then used as queries in Plant Ensembl Database to collect homologues in each rice species and sub-species. Gene, cDNA and putative promoter sequences of eleven rice species, *Oryza barthii, Oryza brachyantha, Oryza glaberrima, Oryza glumipatula, Oryza longistaminata, Oryza meridionalis, Oryza punctata, Oryza rufipogon, Oryza sativa Indica Group, Oryza sativa Japonica Group, Oryza nivara* were retrieved for further use. The information on NLP homologues, the chromosome localization, protein Length (aa) and molecular weight (kDa) are compiled as **(**Supplementary Table 6**)**. Promoter sequence (1000 bp upstream of 5’ UTR) were analysed for the presence of *cis*-regulatory elements using PLANTCARE (http://bioinformatics.psb. ugent.be/web tools/plantcare/html/). Total 95 putative promoter elements were found (including few unnamed elements) in eleven Rice species. Putative promoter elements (RE) including Abscisic acid responsive element (ABA RE), Auxin RE, Light RE, Anaerobic RE, Gibberellin RE, drought RE, salicylic acid RE, methyl jasmonate (MeJA) RE were identified, REs related to defence and stress responsiveness, circadian control, cell cycle regulation, in seed-specific regulation were also found. Light responsive elements were most represented followed by ABA RE. *NLPs* bind to NREs and regulate expression of N- metabolism and signaling genes in response to external cues **(**Supplementary Table 7**)**.

### In *silico* Expression analysis of *NLP*_*S*_

To infer the regulatory role of NLPs in rice development and stress response the expression potential was retrieved from Genevestigator. Expression potential is the normalized expression value for a gene across all experiments available in the database, and the darkest red color represents the “maximum” level of expression for the given probe.

Expression of five *NLP*s (except *NLP*6) in 22 anatomical parts and six *NLP*s in 20 parts were analysed *in silico* in microarray and mRNA seq data (Supplementary Fig. 5). High expression in Flag leaf, shoot and callus. Tissue specificity of expression implies role in development and coordination of developmental triggers. Most of the *NLPs* showed moderate to high levels of expression in different developmental stages of rice. High expression of *NLP*s coincides with early vegetative stage, early and late reproductive phases. Stage specificity of expression implies role in crop establishment and reproductive success in response to nutritional and environmental cues. Stress responsive expression pattern of *NLPs* were analysed using transcriptome data of nutrient level, hormones drought, cold and salt stresses (Supplementary Figs. 5–7). Expression of five *NLPs* (except *NLP6*) in were differentially regulated in response to ABA, salicylic acid and Trans-zeatin treatments (P ≤ 0.05). The presence of the respective putative promoter elements in *NLP* promoter region reinstates the expression data. The hormone responsive expression also indicates the role of *NLPs* in sensing and transducing hormone signals to modulate downstream metabolism. Low (0.5 mM) and high (2 mM) ammonium and low P up- regulate *NLP* expression. Expression levels of six *NLPs* were differentially regulated in response to cold, heat and drought treatments (P ≤ 0.05) **(**Fig. 4**)**. The presence of the respective putative promoter elements in *NLP* promoter region reinstates the expression data. The stress responsive expression also indicates the regulation of N metabolism by environmental stress. Expression levels of six *NLPs* were differentially regulated in response to imbibition, early plant growth and seed deterioration treatments (P ≤ 0.05). The imbibition and seedling specific expression of *NLPs* correlate with the nitrate signalling mediated degradation of ABA and developmental changes as reported in model plants.

### Expression of nitrogen metabolism and *NLP* genes in rice genotypes with contrasting NUE

To further validate and analyze the expression of *NLPs* and other candidate genes, by qRT-PCR, experiments were conducted at seedling stage in hydroponics, in the selected four rice genotypes. Both mRNA expression (/UBQ) and relative expression w.r.to T3 as calibrator is presented. Expression of assimilation genes is represented as Fig. 5a–d. Expression analysis of *OsNIA1, OsNIR, OsGS1.1, OsGS1.2, OsGS2, OsNADH-GOGAT, OsFd-GOGAT* and *OsGDH* were studied in the leaf and root tissues of rice seedlings subjected to treatments T1, T2 and T3 for 24 h. Relative fold expression was computed by keeping T3 treatment (leaf or root) as calibrator in all the genotypes. N assimilatory gene expression was significantly higher in T1compared T2 irrespective of tissues. Relative mRNA expression of N assimilation genes showed conclusive trends. Genotype IR-3-1-1 showed high N assimilatory gene expression followed by APO and NL-42. PB1 showed very low expression of *OsGS1.1, OsGS2, OsGDH and OsNADH-GOGAT*. Plant growth under different N forms most notably impacted gene expression of N assimilation genes in leaves of rice plants. The transcription of primary nitrate assimilation genes, *OsNIA1 and OsNIR, OsGS2* were specifically induced by nitrate supply (T1). Comparison of response to nitrate availability, ammonia supply (T2) also revealed a conspicuous re-programming of N assimilation, for example in IR-3-1-1, the transcript abundance of ammonia assimilation genes, *OsGS1.2, OsNADH-GOGAT and OsGDH* were up regulated.

Expression analysis of *LATS* tansporters *OsNRT1.1B, OsNRT1.2, OsNRT1.5* and *OsNRT1.7* was studied in the root tissues of rice seedlings subjected to treatments T1, T2 and T3 for 24 hrs **(**Fig. 6a–d). mRNA expression (/UBQ) as well as relative fold expression depicts nitrate meditaed (T1) induction of LATS expression. The expression of all the *LATS* genes studied were highest in IR-3-1-1, most importantly, it showed highest expression of *OsNRT1.1B* gene which is one of the rice homologues of *AtNRT1.1* and also plays a role in nitrate uptake, translocation and signaling in rice. PB1 also showed comparatively higher expression of LATS except in the case of *OsNRT1.1B*.

Expression analysis of all 6 *NLPs* were also studied in leaf and root tissues of rice seedlings subjected to treatments T1, T2 and T3 for 24 hrs **(**Fig. 7a–e**)**. Expression of *NLP1* in shoots showed no or negligible expression at 24 h, whereas in roots the expression was up regulated by T1 and T3 treatments. Time course expression analysis of *NLP1* in IR-3-1-1 after 0.5, 2, 24 and 72 hr of exposure to T1 treatment showed that *NLP1* expression was maximum in roots after 30 min exposure to nitrate, and thus *NLP1* may have probable potential role in primary nitrate response **(**Fig. 7e**)**. This kind of temporal variation in expression can be expected in other *NLPs* also. In general, *NLPs* were expressed more in roots. as depicted in Fig. 7a and heatmap Fig. 7b, mRNA expression of NLPs were highest in response to by N deficiency (T3) followed by nitrate treatment (T1), in root tissues. However there were genotypic differences in the pattern of gene expression, for example IR-3-1-1 did not follow upregulation in expression of *NLP* 2/4/5 by N deficiency. Relative fold expression (Fig. 7c,d) depicts that T1 treatment resulted in highest expression of *NLPs*, indicating nitrate regulated expression of *NLPs*. Genotype IR-3-1-1 showed a general up regulation of *NLP* expression which is correlated with higher uptake gene transcript abundance. *NLP3* and *NLP4* expression were highly up regulated in roots of IR-3-1-1 and NL-42 plants receiving T1 treatment.

As the data on N responsive expression of genes was is limited in Genevestigator, we searched NCBI GEO for relevant experiments **(**Fig. 8a,b**)**. Expression data of experiments GSE61370 (rice roots exposed to 0.005 and 1 mM NH_4_Cl for 10 days) and GSE66807 (low N adapted rice plants (0.3 mM NO_*3*_^−^) transferred to normal (high, 3 mM NO_3_^−^) N condition (induction treatment) and plants grown with normal N conditions moved to a low N system (reduction), were downloaded from NCBI-GEO (https://www.ncbi.nlm.nih.gov/gds) and analysed to study *NLP* gene expression. In agreement with our experimental results, expression of *OsNLP3* and *OsNLP4* were responsive to N availability, though the pattern showed by *OsNLP3* was more consistent.

Protein interaction networks of the NLP proteins were predicted by STRING bioinformatics tool (Supplementary Fig. 8). Among all the NLPs, OsNLP1, OsNLP3 and OsNLP4 showed potential interactions with annotated putative target proteins from the rice genome. The cut-off for the protein interaction networks were,2.31e-25 for PB1 NLP1, 1.05e-23 for PB1 NLP3, 1.94e-18 for PB1 NLP4 respectively. OsNLP1 displayed major interaction with, Putative transcription factor PCF3 (Os01g0924400), Protein kinase, domain containing protein, Nitrate transporter NTL1. CBL-interacting protein kinase 8, Putative nodulation receptor kinase etc. OsNLP3 showed potential interactions with Nitrate/chlorate transporter, Putative leucine zipper protein (Os10g0562700), High-affinity nitrate transporter 2.1, Nitrate/chlorate transporter (Os10g0554200), High affinity nitrate transporter (Os02g0112100), Nitrite reductase etc. OsNLP4 has potential interactions with, protein kinase (Os10g0571300), Transcription factor-like (Os02g0739700) protein; Myb-like DNA-binding domain containing protein (Os12g0238000 protein), Leucine-rich repeat transmembrane protein kinase (Os10g0389800), Growth-regulating factor 8 etc. Iinteraction network involving Os NLP3, OsJ_19340 and OS10T0554200-02 (Nitrate/chlorate transporter), *NRT2.1* (*HATS* nitrate transporter 2.1) and nitrite reductase provide evidence that *NLP3* act as central regulator associated with N responses in coordination with *NRT1.1B* and *NRT1.1 A*. Summary of the gene expression regulation by N in rice plants is represented as a schematic model in Fig. 9.

## Discussion

Nitrogen is the most important mineral nutrient essential for crop production and al also function as a nutrient signal determining plant growth and metabolism^38^. NUE of cereal crops including rice is approximately 40%, and the loss of 60% applied N incurs economic and environmental shortfalls^39^. NLPs are transcription factors that recognises NRE motifs in the promoter region of nitrate regulated genes^14^. The study aims at 1) analysing rice genotypes for traits determining NUE 2) genome-wide identification, *insilico* and expression analysis of *NLP* genes family in selected rice genotypes.

We found significant variation in N accumulation of culm and grains. Grain N accumulation is a key indicator of NUE and both NUpE and NUtE (uptake or N utilization efficiencies), in crops^40^,^41,1^. The present research also showed considerably higher N uptake by both grain and straw in case of high NUE genotype IR-3-1-1 when compared with low NUE genotype PB1. NUpE is influenced by mass flow of soil water to the root, root morphology, transporter activity on the root surface, timing of N application, and microbial competition^42^. Significant difference was observed in root traits like total root length, total root volume, total root surface area, with N treatments and varieties. Irrespective of genotype, nitrate nutrition (T1) significantly improved the root growth, root surface area and number of fine root hairs in rice. N deficiency also significantly promoted root proliferation. Genotypic variation in root traits was also significant. Root proliferation also has significant correlation with nitrate uptake kinetics. Barber (1984)^43^ reported that rate of nitrate uptake was a critical parameter for nitrate uptake and the plants having higher rate of uptake can use nitrogen more efficiently. Comparison of rate of uptake revealed that most efficient uptake system was in IR-3-1-1 followed by APO when nitrate supply was low. Whereas at higher N supply rate of nitrate uptake was highest in IR-3-1-1 and PB1. Nitrate uptake at high external concentrations are mediated by NRT1/NPF family of nitrate transporters falling into the category of low affinity nitrate transport system (LATS). The fore most member of LATS family in rice is OsNRT1/OsNPF8.9^44^ and transgenic plants overexpressing of *OsNPF8.9* improved N uptake efficiency under high N conditions^45^. Another prominent member of rice LATS family, *OsNPF6.5/OsNRT1.1B* is involverd in root nitrate uptake and translocation to shoot. Specific *OsNRT1.1B* allele present in *indica* rice could impart improvement in yield (by 30-33%) and NUE in *japonica* NILs^46^ under low N conditions. To understand the varietal differences nitrate uptake, expression analysis of LATS transporters NRT1.1B, NRT1.2, NRT1.5 and NRT1.7 was also studied. Interestingly, the expression of all the *LATS* genes studied were highest in IR-3-1-1, contributing to the high uptake rate. The high expression of *NRT1.1B* gene which is one of the rice homologue of *AtNRT1.1* and also plays a role in nitrate uptake, translocation and signaling in rice. PB1 also showed comparatively higher relative fold change w.r.to other genotypes APO and NL-42.

Transcription factors such as *NLPs, TGA, bZIP1, LBDs, TCPs*, and *NAC4* are involved in nitrate signalling in *Arabidopsis*^47^. The altered transcript levels of these genes regulate downstream NO_3_^−^ responsive genes. In *Arabidopsis*, the presence and abundance of nitrate is perceived by *NPF6.3*, in turn activating phospholipase-C to induce a transient increase in cytoplasmic calcium. This oscillation in calcium level induces the expression of *TGA1/TGA4*. Expression of the auxin receptor, *AFB3* is also responsive to nitrate level via a calcium-independent mechanism. *AFB3* up regulates the expression of *NAC4* and *OBP4*. *TabHLH1*, a *bHLH*-type transcription factor gene in wheat, improves tolerance to N deprivation via regulation of nutrient transporter gene transcription^48^. The current understanding of *NLPs* is mainly based on the studies in *Arabidopsis* and legumes. Apart from the bioinformatic analysis^14,49^, functional studies on rice *NLPs* are lacking, except the finding that OsNRT1;1 affect the subcellular localization of OsNLP4^50^.

We found six *NLP*_*S*_ in rice based on protein BLAST for presence of conserved RWP-RK and PB1 domains (RWP-RK. hmm, PF02042; PB1.hmm, PF00564). The genes were named as *NLP1* to *NLP 6* based on similarity to Arabidopsis *NLPs* and previous reports in Rice^14^. All the other *NLPs* are localised to cellular locations other than mitochondria (mTP) and chloroplast (cTP)- cytosolic and or nuclear localisation as reported in *AtNLPs*^17^. The sequence information of 11 rice species, *Oryza barthii, Oryza brachyantha, Oryza glaberrima, Oryza glumipatula, Oryza longistaminata,, Oryza meridionalis, Oryza punctata, Oryza rufipogon, Oryza sativa Indica Group, Oryza sativa Japonica Group, Oryza nivara* were retrieved and the information on *NLP* homologues, the chromosome localization, protein Length (aa) and molecular weight (kDa) were deciphered. Evolutionary analysis of *NLPs* indicated three origins of this gene family, where Group 3 has the most ancestral genes originating from green algae. The well-known *AtNLP6* and *AtNLP7* genes belong to Group 3^23^. In the current study also, rice and Arabidopsis *NLPs* were classified in to 3 groups. Group III consisted of forty two rice (*NLP2, NLP3, NLP5, NLP6*) and four Arabidopsis (*NLP6, NLP7, NLP8 and NLP9*) NLPs. Hence in agreement with recent reports in rice, *OsNLP3* could be the closest homologue of *AtNLP7*. Spatial and temporal expression pattern *NLPs* in rice development and stress response were analysed by using Genevestigator. High expression of *NLPs* coincides with early vegetative stage, early and late reproductive phases. Stage specificity of expression implies role in crop establishment and reproductive success in response to nutritional and environmental cues. Expression of five *NLPs* (except *NLP6*) was differentially regulated in response to ABA, salicylic acid and Trans-zeatin treatments (P ≤ 0.05). The presence of the respective putative promoter elements in *NLP* promoter region reinstates the expression data. The hormone responsive expression also indicates the role of *NLPs* in sensing and transducing hormone signals to modulate downstream metabolism. Expression of six in was differentially regulated in response to imbibition, early plant growth and seed deterioration treatments (P ≤ 0.05). The imbibition and seedling specific expression of *NLPs* correlate with the nitrate signalling mediated degradation of ABA and developmental changes as reported in model plants. In Arabidopsis, nuclear localisation of *NLP8* stimulate seed germination by activating abscisic acid (ABA) catabolic enzyme and reducing seed ABA level in a nitrate dependent manner^51,52^.

A seedling stage hydropincs experiment was conducted to understand N regulated expression changes of *NLP* genes (Fig. 7). Recently it was found that rice NLP1 protein is nuclear localized localizes in nucleus and the gene expression is N regulated unlike *AtNLPs*. Trangenic manipulation of *OsNLP1* expression correlated that higher expression *OsNLP1* enhances NUE, by regulating both N uptake and assimilation, whereas knocking out of *OsNLP1* reduces NUE and yield under low N conditions^53^. Expression of *NLP1* in shoot showed no or negligible expression at 24 hr, however a time course of *NLP1* expression analysis indicated, the maximum expression was observed in roots after 30 min exposure to nitrate. Primary nitrate response (PNR) is the earliest (peaks at 30 min after exposure) nitrate response observed in higher plants, followed by transcriptional up regulation of nitrate assimilation *(NR, NIR)* genes and nitrate transporters and other related genes. Despite intensive efforts to identify components involved in PNR for last two decades^54^, no significant progress was made until recently after the identification of *AtCIPK8*, a CBL interacting protein kinase which regulate low affinity phase of PNR^55^ nitrate modulated primary root growth in *Arabidopsis*. Protein interaction networks predicted by STRING bioinformatics tool (Supplementary Fig. 8) predicts that *OsNLP1* has an interaction network involving *CIPK8*, which along with the expression peak at 30 min of nitrate exposure makes *OsNLP1* a probable candidate for PNR in rice. There is possibility of involvement of other *NLPs* in PNR as temporal variation in expression is expected in other *NLPs* also. In general, *NLP1, 2, 3* and *6* was expressed more in roots (Supplementary Fig. 8). More over recently showed, under different N regimes of field trials consistently showed that loss-of-*OsNLP4* dramatically reduced yield and NUE compared with wild type. In contrast, the *OsNLP4* overexpression lines remarkably increased yield by 30% and NUE by 47% under moderate N level compared with wild type. It clearly showed that OsNLP4 orchestrates the expression of a majority of known N uptake, assimilation and signaling genes by directly binding to the nitrate-responsive *cis*-element in their promoters to regulate their expression by transcriptomic analyses. Moreover, overexpression of OsNLP4 can recover the phenotype of Arabidopsis *nlp7* mutant and enhance its biomass^56^.N deficiency (T3) and Nitrate (T1) treatments resulted in highest expression of *NLPs* – indicating N deficiency vis-a-vis nitrate regulated expression of *NLPs*. Genotype IR-3-1-1 showed a general up regulation of *NLP* expression which is correlated with higher transcript abundance of nitrate uptake genes. *NLP3* expression was highly up regulated in roots of IR-3-1-1 and NL-42 plants receiving T1 treatment.

*OsNLP3* and *OsNLP4* are interesting candidate genes because of the evolutionary relationship, they are the best rice homologue of *AtNLP6* and *AtNLP7*^14^. Recently, it was shown that the in the transgenic lines expressing *OsNRT1.1 A*/*OsNPF6.3* showed higher nuclear retention of *OsNLP3* and *OsNLP4* and thereby enhanced the expression levels nitrate metabolism genes and promoted early maturation in rice^44^. These transgenic lines also had higher economic production and NUE: demonstrating the major role of *OsNRT1.1 A/ OsNPF6.3-OsNLP3/4* in regulating the uptake and assimilation of nitrate to improve crop yields.

Another important candidate is *OsNRT1.1B* which was found to be highly up regulated in high NUE genotype, IR-3-1-1 (Fig. 6). *OsNRT1.1B* was identified by screening genotypes for resistance to chlorate the toxic analogue of nitrate^57^. The *OsNRT1.1B* allele found in *indica* rice is thought to impart high NUE and is part of a critical QTL contributing to NUE divergence between rice subspecies. Transgenic over expression of *OsNRT1.1B* improved grain yield and NUE of *japonica* rice^57^. OsNRT1.1B also has an important role in regulating nitrate signalling and can contribute in regulating NUpE and NUtE, which both contribute to NUE improvement in rice^57^. OsNRT1.1B and OsNRT1.1 A are both functional paralogs of AtNRT1.1 which functions as a sensor to trigger the PNR^58^ and nitrate signaling in Arabidopsis. The increase in transcript abundance of OsNRT1.1B in high NUE genotype also confirms its probable involvement in determining NUpE and NUtE.

As the data on N responsive expression of genes was is limited in Genevestigator, we searched NCBI GEO for relevant experiments (Fig. 8a,b). Expression data of experiments GSE61370 (rice roots exposed to 0.005 and 1 mM NH_4_Cl for 10 days) and GSE66807 (low N adapted rice plants (0.3 mM NO_3_) transferred to normal (high, 3 mM NO_3_) N condition (induction treatment) and plants grown with normal N conditions moved to a low N system (reduction), were downloaded from NCBI-GEO (https://www.ncbi.nlm.nih.gov/gds) and analysed to study *NLP* gene expression. In agreement with our experimental results, expression of *OsNLP3* and *OsNLP4* were responsive to N availability, though the pattern showed by *OsNLP3* was more consistent.

Hu *et al*.^57^ showed that the nitrate sensor NRT1.1B interacts with a phosphate signalling repressor SPX4 which in turn interacts with NLP3. They demonstrated that SPX4 connects nitrate signal perception through NRT1.1B and downstream nitrate response activation via NLP3 in the nitrate signal transduction pathway. Protein interaction networks predicted by STRING bioinformatics tool (Supplementary Fig. 8) predicts that *OsNLP3* has an interaction network involving OsJ_19340 and OS10T0554200-02 (Nitrate/chlorate transporter) and NRT2.1 (HATS nitrate transporter 2.1) provide evidence that *NLP3* act as central regulator associated with N responses in coordination with NRT1.1B and NRT1.1 A.

## Conclusions

Based on physiological analysis of rice germplasm four genotypes with contrasting NUE were identified. The low NUE variety PB1 showed an overall decline in photosynthetic pigment content in both field and hydroponic condition. Based on the activity and expression of N assimilatory and low affinity nitrate uptake (LATS) genes, we found that PB1 being N responsive genotype was efficient in uptake when N availability is high, IR3-1-1 has high uptake efficiency, translating into high NUE. The expression of all the LATS genes studied were highest in IR3-1-1, most importantly, it showed highest expression of *OsNRT1.1B* gene which is one of the rice homologues of *AtNRT1.1* and also plays a role in nitrate uptake, translocation and signaling in rice. Based on insilico analysis, 6 NLP genes (including alternative splice forms 11NLPs) were identified from rice. Insilico expression profiling, chromosomal localization, putative promoter elements, protein localization, co-expression net-work, were also conducted. Expression of NLPs was promoted by nitrate supply as well as N deficiency (*NLP1, NLP4, NLP5*). The effective and coordinated signal transduction network involving the rice homologue of nitrate transceptor OsNRT1.1B, the probable PNR regulator O_S_NLP1 and the master response regulator OsNLP3, a homologue of AtNLP6/7 renders high NUE in rice **(**Fig. 9).

## Supplementary information

Supplementary information.
