# Supplementary material for: Genome wide analysis of NLP transcription factors reveals their role in nitrogen stress tolerance of rice
Source: Sci Rep. 2020 Jun 10;10:9368. doi: 10.1038/s41598-020-66338-6 (PMC7287097; doi:10.1038/s41598-020-66338-6)
Supplement: Supplementary file 1 — Supplementary information. [file 41598_2020_66338_MOESM1_ESM.docx]

| **Sl.No** | **Primer name** | **Sequence** | **Length (bases)** | **GC (%)** | **Tm (^0^C)** | **Product (bp)** | **Sl.No** | **Primer name** | **Sequence** | **Length (bases)** | **GC (%)** | **Tm (^0^C)** | **Product (bp)** |
| --- | --- | --- | --- | --- | --- | --- | --- | --- | --- | --- | --- | --- | --- |
|  | *Os UBQ-F* | CGAGCCTCTGTTCGTCAAGT | 20 | 60.04 | 55.00 | 116 |  | *Os NR1-F* | CCTACTACTAAATTATACGCACCG | 23 | 56.36 | 39.13 | 232 |
|  | *Os UBQ-R* | CTGCTGTCCCACAGGAAACT | 20 | 59.89 | 55.00 |  |  | *Os NR1-R* | CAGGAAGGAATCAACCGCTA | 17 | 59.68 | 70.59 |  |
|  | *Os NLP1-F* | TCAGGGTTTAAGCGGCATGA | 20 | 59.67 | 50.00 | 198 |  | *Os NiR-F* | CAAATCAGTGTTCCGATAGGTAA | 19 | 58.92 | 57.89 | 197 |
|  | *Os NLP1-R* | TGTAGTCGGGCACACTCCT | 19 | 60.23 | 57.89 |  |  | *Os NiR-R* | GGCTGGAGACGGTGGTG | 17 | 59.33 | 70.59 |  |
|  | *Os NLP2-F* | GTACTGCAGCTGTGTTCAGGA | 21 | 60.27 | 52.38 | 173 |  | *Os GS1;1-F* | CCGTCTGTCGGCATTTCTG | 21 | 60.97 | 57.14 | 197 |
|  | *Os NLP2-R* | GGAACTAGGCTGTACCTGCTC | 21 | 59.86 | 57.14 |  |  | *Os GS1;1-R* | GGGATGGGCTTGGGGTC | 22 | 59.58 | 45.45 |  |
|  | *Os NLP3-F* | TTGGCAGGCCAAATGGTAGT | 20 | 59.89 | 50 | 167 |  | *Os GS1;2-F* | GCCCACAGGGACCATACTACT | 19 | 58.92 | 57.89 | 117 |
|  | *Os NLP3-R* | TGTGGGACATACACCAAGGC | 20 | 59.96 | 55 |  |  | *Os GS1;2-R* | CGTTGATGCCACTGATGTTGAT | 16 | 62.65 | 75 |  |
|  | *Os NLP4-F* | CACAATATTTGGATGTCCACGGG | 23 | 59.93 | 47.83 | 161 |  | *Os GS2-F* | AGTATGCGTGAAGATGGAGGAT | 24 | 60.79 | 45.83 | 190 |
|  | *Os NLP4-R* | TGTCAGGTTAACTGCCTGGAG | 21 | 59.65 | 52.38 |  |  | *Os GS2-R* | GCCCCACCCGAATAGAGC | 18 | 60.76 | 66.67 |  |
|  | *Os NLP5-F* | AATGCTCTGCAGTCTGTTCAGT | 20 | 59.96 | 55 | 165 |  | *Os NADH-GOGAT1-F* | TTGCGGTTACAAGACACTCTACTG | 17 | 61.76 | 70.59 | 238 |
|  | *Os NLP5-R* | GGAGCAAACTGGAATCCATGC | 20 | 59.9 | 55 |  |  | *Os NADH-GOGAT1-R* | GCTCCCGTCCCTCCATCA | 25 | 59.36 | 40 |  |
|  | *Os NLP6-F* | GGAGGAGCTGCAGAAACACT | 20 | 59.89 | 55 | 127 |  | *Os Fd-GOGAT-F* | TGGTCTCCGCCCAGCAC | 20 | 53.27 | 40 | 149 |
|  | *Os NLP6-R* | GATCTTGCGAAATGGCCACC | 20 | 60.03 | 55 |  |  | *Os Fd-GOGAT-R* | CAGTTTGTAGGTCAACCGTTATCAT | 19 | 53.73 | 42.11 |  |
|  | *Os NRT1.1B-F* | AGGTTCAAGAAGCTGGTGGG | 20 | 59.94 | 63.26 | 162 |  | *Os GDH2-F* | GGCCATTAACAACACTCATA | 21 | 60.27 | 52.38 | 127 |
|  | *Os NRT1.1B-R* | CATCCTCTGCTTGCCCTTGA | 20 | 60.11 | 55 |  |  | *Os GDH2-R* | ACGCCGATCTATCTTGAAT | 21 | 59.86 | 57.14 |  |
|  | *Os NRT1.5-F* | CTGTCGAGCTACCTGAGCG | 20 | 60.11 | 55 | 172 |  |  |  |  |  |  |  |
|  | *OsNRT1.5-R* | CGACGAACGCCAAGAAGTTG | 20 | 59.97 | 60 |  |  |  |  |  |  |  |  |
|  | *Os NRT1.2-R* | CGGGATGGTGAACGACATGA | 20 | 59.96 | 55 | 177 |  |  |  |  |  |  |  |
|  | *Os NRT1.2-F* | GATGCCTCGTGGAGGATCTG | 20 | 59.76 | 55 |  |  |  |  |  |  |  |  |
|  | *Os NRT1.7-F* | CACTTCCAGCTCTCAAGCCA | 24 | 56.84 | 41.67 | 195 |  |  |  |  |  |  |  |
|  | *Os NRT1.7-R* | TTCGACCTTCTTCGTCGGTC | 20 | 57.31 | 50 |  |  |  |  |  |  |  |  |

**Supplementary table 1**

**Supplementary Table. 2 Sidak's multiple comparisons test for influence of +N and –N on N content and NUE of rice cv. APO, IR 3-1-1, NL-42, PB1**

| **%N** | | | | **NUE (g grain g^-1^ N uptake)** | | | |
| --- | --- | --- | --- | --- | --- | --- | --- |
| **N- - N+** | **Significant?** | **Summary** | **Adjusted P Value** | **N- - N+** | **Significant?** | **Summary** | **Adjusted P Value** |
| **+N-L vs. -N-L** | No | ns | **0.9757** | **APO** | Yes | **** | **<0.0001** |
| **+N-S vs. -N-S** | Yes | *** | **0.0003** | **IR 3-1-1** | Yes | **** | **<0.0001** |
| **+N-G vs. -N-G** | Yes | *** | **0.0002** | **NL - 42** | Yes | **** | **<0.0001** |
|  | | | | **PB 1** | Yes | ** | **0.0091** |

Treatments: +N 120 kg nitrogen, -N without nitrogen; Replication =3;. APO, IR 3-1-1= IR-83929-B-B-291-3-1-1, NL-42= Nerica-L-42, PB 1= PUSA BASMATI-1, NUE=Nitrogen use efficiency..

**Supplementary Table. 3 Sidak's multiple comparisons test for influence of T1, T2 and T3 on root traits of rice cv. APO, IR 3-1-1, NL-42, PB1**

| **Total root length (cm)** | | | | **Total root surface area (cm^2^)** | | | |
| --- | --- | --- | --- | --- | --- | --- | --- |
|  | **Significant?** | **Summary** | **Adjusted P Value** |  | **Significant?** | **Summary** | **Adjusted P Value** |
| **APO** |  | | | **APO** |  | | |
| **T1 vs. T2** | Yes | ** | **0.013** | **T1 vs. T2** | No | ns | **0.0753** |
| **T1 vs. T3** | Yes | ** | **0.013** | **T1 vs. T3** | No | ns | **0.0969** |
| **IR 3-1-1** |  | | | **IR 3-1-1** |  | | |
| **T1 vs. T2** | Yes | ** | **0.013** | **T1 vs. T2** | No | ns | **0.0632** |
| **T1 vs. T3** | Yes | ** | **0.013** | **T1 vs. T3** | No | ns | **0.0521** |
| **NL - 42** |  | | | **NL - 42** |  | | |
| **T1 vs. T2** | Yes | ** | **0.013** | **T1 vs. T2** | No | ns | **0.4093** |
| **T1 vs. T3** | Yes | ** | **0.013** | **T1 vs. T3** | Yes | * | **0.0452** |
| **PB 1** |  | | | **PB 1** |  | | |
| **T1 vs. T2** | Yes | ** | **0.013** | **T1 vs. T2** | Yes | * | **0.0477** |
| **T1 vs. T3** | Yes | ** | **0.013** | **T1 vs. T3** | No | ns | **0.0774** |
| **Average diameter (mm)** | | | | **Root tips** | | | |
|  | **Significant?** | **Summary** | **Adjusted P Value** |  | **Significant?** | **Summary** | **Adjusted P Value** |
| **APO** |  | | | **APO** |  | | |
| **T1 vs. T2** | No | ns | **0.8375** | **T1 vs. T2** | No | ns | **0.6412** |
| **T1 vs. T3** | No | ns | **0.0916** | **T1 vs. T3** | Yes | ** | **0.0017** |
| **IR 3-1-1** |  | | | **IR 3-1-1** |  | | |
| **T1 vs. T2** | No | ns | **0.189** | **T1 vs. T2** | Yes | * | **0.0153** |
| **T1 vs. T3** | No | ns | **0.7012** | **T1 vs. T3** | No | ns | **0.0531** |
| **NL - 42** |  | | | **NL - 42** |  | | |
| **T1 vs. T2** | No | ns | **0.1525** | **T1 vs. T2** | No | ns | **0.7755** |
| **T1 vs. T3** | No | ns | **0.9074** | **T1 vs. T3** | No | ns | **0.1044** |
| **PB 1** |  | | | **PB 1** |  | | |
| **T1 vs. T2** | No | ns | **0.326** | **T1 vs. T2** | Yes | * | **0.0141** |
| **T1 vs. T3** | No | ns | **0.9816** | **T1 vs. T3** | Yes | ** | 0.0024 |
| **T1 vs. T3** | No | ns | **0.9973** | **T1 vs. T3** | Yes | ** | 0.0024 |

Treatments: T1=High nitrate (HN), 6.5mM NO_3_^-^+ 1mM NH_4_^+^, T2=High ammonium (HA), 6.5mM NH_4_^+^+ 1mM NO_3_^-^, T3=75 µM NO_3_^-^ (LN), Low N, 0.12mM NO_3_^-^ + 0.12mM NH_4_^+^; Replication =3;. APO, IR 3-1-1= IR-83929-B-B-291-3-1-1, NL-42= Nerica-L-42, PB 1= PUSA BASMATI-1.

**Supplementary Table. 4 Sidak's multiple comparisons test for influence of T1, T2 and T3 on root traits and rate of nitrate uptake of rice cv. APO, IR 3-1-1, NL-42, PB**

| **Total root volume (cm^3^)** | | | |
| --- | --- | --- | --- |
|  | **Significant?** | **Summary** | **Adjusted P Value** |
| **APO** |  | | |
| **T1 vs. T2** | Yes | ** | **0.008** |
| **T1 vs. T3** | Yes | ** | **0.0038** |
| **IR 3-1-1** |  | | |
| **T1 vs. T2** | No | **V1 vs V2** | **0.1626** |
| **T1 vs. T3** | No | ns | **0.1318** |
| **NL - 42** |  | | |
| **T1 vs. T2** | No | ns | **0.417** |
| **T1 vs. T3** | No | ns | **>0.9999** |
| **PB 1** |  | | |
| **T1 vs. T2** | No | ns | **0.4792** |
| **T1 vs. T3** | No | ns | **0.9973** |
|  | | | |
| **Rate of nitrate uptake** | | | |
|  | **Significant?** | **Summary** | **Adjusted P Value** |
| **100µM** | **Yes** | ***8** | **0.0313** |
| **1mM** | **Yes** | ****** | **0.005** |

Treatments: T1=High nitrate (HN), 6.5mM NO_3_^-^+ 1mM NH_4_^+^, T2=High ammonium (HA), 6.5mM NH_4_^+^+ 1mM NO_3_^-^, T3=75 µM NO_3_^-^ (LN), Low N, 0.12mM NO_3_^-^ + 0.12mM NH_4_^+^; Replication =3;. APO, IR 3-1-1= IR-83929-B-B-291-3-1-1, NL-42= Nerica-L-42, PB 1= PUSA BASMATI-1.

**Supplementary Table. 5 Adjusted P values computed from one-way anova analysis of gene expression data**

|  | Significant? | Summary | Adjusted P Value |  | Significant? | Summary | Adjusted P Value |
| --- | --- | --- | --- | --- | --- | --- | --- |
| *Os NLP1* | Yes | ** | 0.012 | *Os NR1* | Yes | ** | 0.0013 |
| *Os NLP2* | *Yes* | * | 0.048 | *Os NiR* | *Yes* | ** | 0.013 |
| *Os NLP3* | Yes | ** | 0.018 | *Os GS1;1* | Yes | ** | 0.046 |
| *Os NLP4* | Yes | * | 0.047 | *Os GS1;2* | Yes | * | 0.019 |
| *Os NLP5* | Yes | ** | 0.011 | *Os GS2* | Yes | ** | 0.013 |
| *Os NLP6* | *Yes* | * | 0.018 | *Os GS2* | *Yes* | ** | 0.012 |
| *Os NRT1.1B* | Yes | ** | 0.013 | *Os NADH-GOGAT1* | Yes | * | 0.019 |
| *Os NRT1.5* | Yes | * | 0.02 | *Os Fd-GOGAT* | Yes | ** | 0.013 |
| *Os NRT1.2* | Yes | * | 0.04 | *Os GDH2* | Yes | ** | 0.0034 |
| *Os NRT1.7* | *Yes* | ** | 0.014 |  |  |  |  |

**Supplementary Table 6** Structural details of *NLP* homologues in *Oryza* spp

| ***Oryza spp*** | | | | | |
| --- | --- | --- | --- | --- | --- |
| **Gene Id** | | **Chromosome** | **Protein Length (aa)** | **Name** | **Mwt (kDa)** |
| ***Oryza sativa Indica Group*** | | | | | |
| *BGIOSGA011521* | 3 | | 919 | *OsiNLP1* | 101,847.80 g/mol |
| *BGIOSGA016686* | 4 | | 936 | *OsiNLP2* | 101,515.87 g/mol |
| *BGIOSGA003095* | 1 | | 866 | *OsiNLP3* | 95,244.71 g/mol |
| *BGIOSGA031219* | 9 | | 871 | *OsiNLP4* | 95,795.70 g/mol |
| *BGIOSGA034185* | 11 | | 948 | *OsiNLP5* | - |
| *BGIOSGA007168* | 2 | | 719 | *OsiNLP6* | - |
| ***Oryza sativa Japonica Group*** | | | | | |
| *Os03g0131100* | 3 | | 942 | *OsjNLP1* | 104,616.55 g/mol |
| *Os04g0495800, Os04t0495800-02* | 4 | | 936,893 | *OsjNLP2* | 101,635.01 g/mol, 97,222.21 g/mol |
| *Os01g0236700* | 1 | | 938 | *OsjNLP3* | 102,438.72 g/mol |
| *Os09g0549450* | 9 | | 842 | *OsjNLP4* | 92,593.99 g/mol |
| *Os11g0264300* | 11 | | 281,279 | *OsjNLP5* | - |
| *Os02g0136000* | 2 | | 728 | *OsjNLP6* | - |
| ***Oryza nivara*** | | | | | |
| *ONIVA03G02090* | 3 | | 942 | *OnNLP1* | 104,555.50 g/mol |
| *ONIVA04G15630* | 4 | | 930 | *OnNLP2* | 100,909.22 g/mol |
| *ONIVA01G11240* | 1 | | 938 | *OnNLP3* | 102,412.64 g/mol |
| *ONIVA09G19860* | 9 | | 837 | *OnNLP4* | 92,198.53 g/mol |
| *ONIVA11G08980* | 11 | | 1011,984,956,952,951,924,923,916,896,838,65 | *OnNLP5* | - |
| *ONIVA02G02790,ONIVA02G02780* | 2 | | 753,724,716,306 | *OnNLP6* | - |

| ***Oryza spp*** | | | | |
| --- | --- | --- | --- | --- |
| **Gene Id** | **Chromosome** | **Protein Length (aa)** | **Name** | **Mwt (kDa)** |
| ***Oryza punctata*** | | | | |
| *OPUNC03G02270* | 3 | 995 | *OpNLP1* | 110,081.76 g/mol |
| *-* | - | - | *OpNLP2* | - |
| *OPUNC01G08520* | 1 | 948 | *OpNLP3* | 103,712.09 g/mol |
| *OPUNC09G17100* | 9 | 842 | *OpNLP4* | 92,743.21 g/mol |
| *OPUNC11G08590* | 11 | 947,894,866 | *OpNLP5* | - |
| *OPUNC02G02310,OPUNC02G02320* | 2 | 253,628 | *OpNLP6* | - |
| ***Oryza rufipogon*** | | | | |
| *ORUFI03G02230* | 3 | 942 | *OrNLP1* | 104,555.50 g/mol |
| *ORUFI04G18750* | 4 | 930 | *OrNLP2* | 100,958.21 g/mol |
| *ORUFI01G09470* | 1 | 938 | *OrNLP3* | 102,438.72 g/mol |
| *ORUFI09G20290* | 9 | 837 | *OrNLP4* | 92,260.56 g/mol |
| *ORUFI11G09500* | 11 | 967,910,885,882 | *OrNLP5* | - |
| *ORUFI02G02860, ORUFI02G02850* | 2 | 724,306 | *OrNLP6* | - |

| ***Oryza spp*** | | | | |
| --- | --- | --- | --- | --- |
| **Gene Id** | **Chromosome** | **Protein Length (aa)** | **Name** | **Mwt (kDa)** |
| ***Oryza barthii*** | | | | |
| *OBART03G02480* | 3 | 850 | *ObNLP1* | 94,578.46 g/mol |
| *OBART04G17320* | 4 | 930 | *ObNLP2* | 100,935.17 g/mol |
| *OBART01G08660* | 1 | 866 | *ObNLP3* | 95,214.69 g/mol |
| *OBART09G18730* | 9 | 842 | *ObNLP4* | 92,625.00 g/mol |
| *OBART11G09000* | 11 | 932 | *ObNLP5* | - |
| *OBART02G02910,OBART02G03000* | 2 | 306,218 | *ObNLP6* | - |
| ***Oryza brachyantha*** | | | | |
| *OB03G12460* | 3 | 898 | *ObrNLP1* | 101,031.34 g/mol |
| *OB04G24560* | 4 | 935 | *ObrNLP2* | 102,059.44 g/mol |
| *OB01G18870* | 1 | 751 | *ObrNLP3* | 82,377.24 g/mol |
| *OB09G25360* | 9 | 841 | *ObrNLP4* | 92,453.66 g/mol |
| *OB11G17130* | 11 | 596 | *ObrNLP5* | - |
| *-* | - | - | *ObrNLP6* | - |
| ***Oryza glaberrima*** | | | | |
| *ORGLA03G0022700* | 3 | 942 | *OgNLP1* | 104,561.60 g/mol |
| *ORGLA04G0144400* | 4 | 936 | *OgNLP2* | 101,563.92 g/mol |
| *ORGLA01G0077900* | 1 | 941 | *OgNLP3* | 102,755.98 g/mol |
| *ORGLA09G0149200* | 9 | 842 | *OgNLP4* | 92,596.99 g/mol |
| *ORGLA11G0080000* | 11 | 881 | *OgNLP5* | - |
| *ORGLA02G0027800* | 2 | 671 | *OgNLP6* | - |

| ***Oryza spp*** | | | | |
| --- | --- | --- | --- | --- |
| **Gene Id** | **Chromosome** | **Protein Length (aa)** | **Name** | **Mwt (kDa)** |
| ***Oryza glumipatula*** | | | | |
| *OGLUM03G02410* | 3 | 945 | *OglNLP1* | 104,932.87 g/mol |
| *OGLUM04G17360* | 4 | 929 | *OglNLP2* | 100,855.22 g/mol |
| *OGLUM01G09910* | 1 | 942 | *OglNLP3* | 102,799.00 g/mol |
| *OGLUM09G19240* | 9 | 841,888 | *OglNLP4* | 92,487.83 g/mol |
| *OGLUM11G08660* | 11 | 901,886,864,858 | *OglNLP5* | - |
| *OGLUM02G02830,OGLUM02G02840* | 2 | 306,721 | *OglNLP6* | - |
| ***Oryza longistaminata*** | | | | |
| *KN538737.1_FG044* | [Scaffold KN538737.1: 312,014-314,738](http://plants.ensembl.org/Oryza_longistaminata/Location/View?db=core;g=KN538737.1_FG044;r=KN538737.1:312014-314738;t=KN538737.1_FGT044;tl=cTTVHM8lTdpGtEQe-19168297-792810155) | 677 | *OlNLP1* | 75,568.07 g/mol |
| *KN539504.1_FG009* | [Scaffold KN539504.1: 22,300-28,041](http://plants.ensembl.org/Oryza_longistaminata/Location/View?db=core;g=KN539504.1_FG009;r=KN539504.1:22300-28041;t=KN539504.1_FGT009;tl=5lNAOT9W3ipDE8J9-19168317-792810293) | 933 | *OlNLP2* | 101,790.94 g/mol |
| *KN539047.1_FG007* | [Scaffold KN539047.1: 9,207-13,201](http://plants.ensembl.org/Oryza_longistaminata/Location/View?db=core;g=KN539047.1_FG007;r=KN539047.1:9207-13201;t=KN539047.1_FGT007;tl=VrM9h8zjvM1jZbqH-19168336-792810522) | 842 | *OlNLP3* | 92,901.11 g/mol |
| *KN539310.1_FG011* | [Scaffold KN539310.1: 47,394-51,821](http://plants.ensembl.org/Oryza_longistaminata/Location/View?db=core;g=KN539310.1_FG011;r=KN539310.1:47394-51821;t=KN539310.1_FGT011;tl=BxwI0iHIvrwpAU7l-19168364-792811343) | 865 | *OlNLP4* | 95,004.69 g/mol |
| *KN539017.1_FG012* | [Scaffold KN539017.1: 111,073-117,852](http://plants.ensembl.org/Oryza_longistaminata/Location/View?db=core;g=KN539017.1_FG012;r=KN539017.1:111073-117852;t=KN539017.1_FGT012;tl=1QllSijHXhcxMtgV-19169657-793468611) | 932 | *OlNLP5* | - |
| *KN540047.1_FG004* | [Scaffold KN540047.1: 59,455-62,313](http://plants.ensembl.org/Oryza_longistaminata/Location/View?db=core;g=KN540047.1_FG004;r=KN540047.1:59455-62313;t=KN540047.1_FGT004;tl=ZkUwpS52HUUvtrIB-19169670-793490688) | 587 | *OlNLP6* | - |
| ***Oryza meridionalis*** | | | | |
| *OMERI03G02340* | 3 | 986 | *OmNLP1* | 109,869.06 g/mol |
| *OMERI04G14630* | 4 | 930 | *OmNLP2* | 100,895.32 g/mol |
| *OMERI01G08690* | 1 | 943 | *OmNLP3* | 103,068.42 g/mol |
| *OMERI06G03620* | 6 | 842,899,853,870,824 | *OmNLP4* | 93,840.42 g/mol |
| *OMERI11G08000,OMERI11G08010* | 11 | 951,1058,936,996,62,883,987,974,885,682,536 | *OmNLP5* | - |
| *OMERI02G03540,OMERI02G03550* | 2 | 306,747 | *OmNLP6* | - |

**Supplementary Table 7** Comparison of regulatory elements in of *NLP* promoter regions in *Oryza* spp ( 1, 2, 3, 4, 5, 6 respectively *NLP1, NLP2, NLP3, NLP4, NLP5, NLP6*)

| **S.no** | **Cis elements** | **Oba** | **Obr** | **Ogla** | **Oglu** | **Ol** | **Om** | **On** | **Op** | **Or** | **Osi** | **Osj** | **Function** |
| --- | --- | --- | --- | --- | --- | --- | --- | --- | --- | --- | --- | --- | --- |
| **1** | **3-AF1 binding site**  (TAAGAGAGGAA) | **6** | **0** | **6** | **6** | **0** | **0** | **6** | **1,6** | **6** | **0** | **0** | **light responsive element** |
| **2** | **A-box**  (CCGTCC) | **1,4** | **2** | **1** | **1,6** | **1,6** | **1** | **1,5,6** | **1,5,6** | **1,5,6** | **1,4,5,** | **1,3,4,5,6** | **cis-acting regulatory element** |
| **3** | **AAAC-motif**  (CAATCAAAACCT) | **0** | **0** | **0** | **0** | **0** | **0** | **0** | **4** | **0** | **0** | **2** |  |
| **4** | **AAGAA-motif**  (GAAAGAA) | **5** | **1,2,4** | **5** | **3,5** | **3,5** | **0** | **5** | **0** | **5** | **5,6** | **3,5** |  |
| **5** | **ABRE**  (TACGGTC) | **2,4,6** | **1,2,5** | **2,3,5,6** | **4,5,6** | **4,5,6** | **2,5,6** | **1,3,4,5** | **1,3,4,6** | **1,4,5,6** | **1,3,4,5** | **1,2,3,5,6** | **cis-acting element involved in the abscisic acid responsiveness** |
| **6** | **ABRE2**  (CCACGTGG) | **5** | **0** | **5** | **5** | **5** | **0** | **5** | **0** | **0** | **0** | **0** |  |
| **7** | **ABRE3a**  (TACGTG) | **0** | **0** | **0** | **4,5** | **4** | **0** | **3,4** | **0** | **4** | **0** | **3** |  |
| **8** | **ABRE4** (CACGTA) | **0** | **0** | **0** | **4,5** | **4** | **0** | **3,4** | **0** | **4** | **0** | **3** |  |
| **9** | **ACE**  (GCGACGTACC) | **1,6** | **2** | **1,6** | **1** | **6** | **1** | **1** | **1** | **1,2,5** | **1 S** | **1** | **cis-acting element involved in light responsiveness** |
| **10** | **AC-I**  ((T/C)C(T/C)(C/T)ACC(T/C)ACC) | **2** | **0** | **2** | **0** | **0** | **0** | **0** | **0** | **0** | **0** | **0** |  |
| **11** | **AC-II**  (CCACCAACCCCC) | **2** | **0** | **2** | **0** | **0** | **0** | **0** | **0** | **0** | **0** | **0** |  |
| **12** | **ACA-motif**  (AATCACAACCATA) | **0** | **0** | **0** | **0** | **0** | **3** | **0** | **0** | **0** | **0** | **0** | **part of gapA in (gapA-CMA1) involved with light responsiveness** |
| **13** | **AE-box**  (AGAAACAA) | **6** | **0** | **6** | **6** | **6** | **0** | **6** | **6** | **6** | **0** | **6** | **part of a module for light response** |

| **S.no** | **Cis elements** | **Oba** | **Obr** | **Ogla** | **Oglu** | **Ol** | **Om** | **On** | **Op** | **Or** | **Osi** | **Osj** | **Function** |
| --- | --- | --- | --- | --- | --- | --- | --- | --- | --- | --- | --- | --- | --- |
| **14** | **ARE**  (AAACCA) | **1,2,4,5,6** | **2,3,4,5** | **1,2,4,5,6** | **1,2,4,5,** | **1,2,4,5,6** | **1,4,** | **1,2,3,4,5,6** | **3,4,5** | **1,2,3,4,5,6** | **1,2,4,5,6** | **1,2,3,4,5,6** | **cis-acting regulatory element essential for the anaerobic induction** |
| **15** | **as-1**  (TGACG) | **2,3,5,6** | **1,3** | **2,5,6** | **2,3,5,6** | **2,3,5,6** | **2,5,** | **2,5,6** | **1,5,6** | **2,5,6** | **2,5,6** | **1,2,5,6** |  |
| **16** | **AT-rich sequence**  (TAAAATACT) | **1** | **0** | **0** | **0** | **0** | **0** | **0** | **0** | **0** | **0** | **2** | **element for maximal elicitor-mediated activation (2copies)** |
| **17** | **ATC-motif**  (AGTAATCT) | **1** | **0** | **0** | **0** | **0** | **0** | **0** | **0** | **0** | **5,6** | **0** | **part of a conserved DNA module involved in light responsiveness** |
| **18** | **ATCT-motif**  (AATCTAATCC) | **1,6** | **2** | **1,6** | **1,** | **0** | **0** | **0** | **0** | **0** | **0** | **0** | **part of a conserved DNA module involved in light responsiveness** |
| **19** | **AT~TATA-box**  (TATATA) | **1,3,6** | **2,4,5** | **1,6** | **1,3,4,6** | **6** | **1,5,6** | **1,3,4,6** | **6** | **1,3,4,5,6** | **1,6** | **1,3,6** |  |
| **20** | **AT1-motif**  (AATTATTTTTTATT) | **0** | **0** | **0** | **0** | **4** | **0** | **0** | **0** | **0** | **0** | **0** | **part of a light responsive module** |
| **21** | **AuxRR-core**  (GGTCCAT) | **0** | **0** | **0** | **0** | **0** | **0** | **0** | **0** | **0** | **5** | **5,6** | **cis-acting regulatory element involved in auxin responsiveness** |
| **22** | **Box 4**  (ATTAAT) | **2,4,6** | **1,2,4** | **1,2,6** | **1,2,4,6** | **1,4,6** | **2,3,6** | **1,2,4,6** | **1,4** | **1,2,4,6** | **1,2,4,6** | **1,2,4,6** | **part of a conserved DNA module involved in light responsiveness** |
| **23** | **Box II**  (ACACGTTGT) | **0** | **0** | **0** | **0** | **0** | **0** | **1** | **0** | **1** | **1** | **1** | **part of a light responsive element** |
| **24** | **box S**  (AGCCACC) | **0** | **0** | **0** | **0** | **0** | **0** | **0** | **0** | **0** | **0** | **6** |  |
| **25** | **CAAT-box**  (CAAAT) | **1,2,3,4,5,6** | **1,2,3,4,5** | **1,2,3,4,5,6** | **1,2,3,4,5,6** | **1,2,3,4,5,6** | **1,2,3,4,5,6** | **1,2,3,4,5,6** | **1,3,4,5,6** | **1,2,3,4,5,6** | **1,2,3,4,5,6** | **1,2,3,4,5,6** | **common cis-acting element in promoter and enhancer regions** |

| **S.no** | **Cis elements** | **Oba** | **Obr** | **Ogla** | **Oglu** | **Ol** | **Om** | **On** | **Op** | **Or** | **Osi** | **Osj** | **Function** |
| --- | --- | --- | --- | --- | --- | --- | --- | --- | --- | --- | --- | --- | --- |
| **26** | **CAT-box**  (GCCACT) | **1,3,4** | **3,4** | **1,4** | **1** | **2** | **3** | **1** | **0** | **1,3,** | **1,6** | **1,2,3,4,5** | **cis-acting regulatory element related to meristem expression** |
| **27** | **CCGTCC motif**  (CCGTCC) | **1,4** | **2** | **1,** | **1,6** | **1,6** | **1,** | **1,5,6** | **1,5,6** | **1,5,6** | **1,4,5,6** | **1,3,4,5,6** |  |
| **28** | **CCGTCC-box**  (CCGTCC) | **1,4** | **2** | **1,** | **1,6** | **1,6** | **1,** | **1,5,6** | **1,5,6** | **1,5,6** | **1,4,5,6** | **1,3,4,5,6** |  |
| **29** | **CGTCA-motif**  (CGTCA) | **2,3,5,6** | **1,3,** | **2,5,6** | **2,3,5,6** | **2,3,5,6** | **2,5** | **2,5,6** | **1,5,6** | **2,6** | **2,5** | **1,2,6** | **cis-acting regulatory element involved in the MeJA-responsiveness** |
| **30** | **CCAAT-box**  (CAACGG) | **4,5,6** | **0** | **5,6** | **4,5** | **4,5** | **5** | **4,5,6** | **4** | **4,5,6** | **4** | **6** | **MYBHv1 binding site** |
| **31** | **CAG-motif**  (GAAAGGCAGAC) | **0** | **3** | **0** | **0** | **0** | **0** | **0** | **0** | **0** | **0** | **0** | **part of a light response element** |
| **32** | **CTAG-motif** (ACTAGCAGAA) | **0** | **0** | **0** | **3,4** | **4** | **4** | **4** | **0** | **4** | **0** | **0** |  |
| **33** | **chs-CMA1a**  (TTACTTAA) | **0** | **0** | **0** | **3,4** | **0** | **0** | **0** | **0** | **0** | **0** | **6** | **part of a light responsive element** |
| **34** | **C-box**  (ACGAGCACCGCC) | **0** | **0** | **0** | **0** | **0** | **0** | **0** | **1** | **0** | **0** | **0** | **cis-acting regulatory element involved in light responsiveness** |
| **35** | **Circadian**  (CAAAGATATC) | **0** | **0** | **0** | **0** | **0** | **0** | **0** | **6** | **0** | **0** | **1** | **cis-acting regulatory element involved in circadian control** |
| **36** | **chs-CMA2a**  (TCACTTGA) | **0** | **0** | **0** | **0** | **0** | **0** | **0** | **0** | **3** | **0** | **0** | **part of a light responsive element** |
| **37** | **DRE core**  (GCCGAC) | **1,5** | **1,3** | **1,5,6** | **1,5,6** | **5** | **5** | **1,5,6** | **1** | **6** | **1,3,5,6** | **1,2,3,5,6** |  |
| **38** | **DRE1**  (ACCGAGA) | **0** | **4** | **0** | **0** | **0** | **0** | **3** | **0** | **0** | **0** | **0** |  |
| **39** | **Doct**  (CACGGATC) | **0** | **0** | **0** | **0** | **0** | **1** | **0** | **3,** | **0** | **0** | **0** |  |

| **S.no** | **Cis elements** | **Oba** | **Obr** | **Ogla** | **Oglu** | **Ol** | **Om** | **On** | **Op** | **Or** | **Osi** | **Osj** | **Function** |
| --- | --- | --- | --- | --- | --- | --- | --- | --- | --- | --- | --- | --- | --- |
| **40** | **ERE**  (ATTTTAAA) | **3,4** | **2,4,5** | **3,4** | **4** | **4** | **1,4** | **3,4,6** | **4,6** | **3,6** | **5,6** | **3,4,6** |  |
| **41** | **E2Fb**  (TTTGCCGC) | **0** | **3** | **0** | **0** | **0** | **0** | **0** | **0** | **0** | **0** | **0** |  |
| **42** | **F-box**  (CTATTCTCATT) | **0** | **0** | **0** | **6** | **0** | **6** | **0** | **6** | **6** | **0** | **0** |  |
| **43** | **G-box**  (CACGAC) | **1,3,5,6** | **1,2,3,5** | **1,3,5,6** | **1,4,5,6** | **4,5** | **1,2,5,6** | **1,3,4,5,** | **1,3,4,6** | **1,4,5,6** | **1,3,4,5,** | **1,2,3,5** | **cis-acting regulatory element involved in light responsiveness** |
| **44** | **GC-motif**  (CCCCCG) | **1,2** | **1,3** | **1,2,6** | **1,2,6** | **1,2,6** | **5,6** | **1,2,6** | **6** | **1,2,6** | **1,2,3,5** | **1,2,3,6** | **enhancer-like element involved in anoxic specific inducibility** |
| **45** | **GA-motif**  (ATAGATAA) | **3** | **0** | **0** | **0** | **0** | **3** | **3** | **0** | **3** | **0** | **3** | **part of a light responsive element** |
| **46** | **GT1-motif**  (GGTTAA) | **3,6** | **0** | **3,6** | **6** | **3,6** | **2,6** | **3,6** | **6** | **3,6** | **3,4,6** | **3,6** | **light responsive element** |
| **47** | **GATA-motif**  (GATAGGG) | **6** | **0** | **6** | **6** | **6** | **5** | **0** | **5,** | **2,5** | **0** | **2,3** | **part of a light responsive element** |
| **48** | **GCN4_motif**  (TGAGTCA) | **6** | **0** | **6** | **6** | **3** | **0** | **0** | **0** | **0** | **0** | **0** | **cis-regulatory element involved in endosperm expression** |
| **49** | **GARE-motif**  (TCTGTTG) | **0** | **5** | **0** | **6** | **2,3** | **6** | **6** | **6** | **6** | **0** | **2,5** | **gibberellin-responsive element** |
| **50** | **Gap-box**  (CAAATGAA(A/G)) | **0** | **0** | **0** | **0** | **4** | **0** | **0** | **0** | **0** | **0** | **0** | **Apart of a light responsive element** |
| **51** | **GTGGC-motif**  (GATTCTGTGGC) | **0** | **0** | **0** | **0** | **0** | **0** | **0** | **1** | **0** | **0** | **0** | **part of a light responsive element** |
| **52** | **GATT-motif**  (CTCCTGATTGGA) | **0** | **0** | **0** | **0** | **0** | **0** | **0** | **0** | **0** | **0** | **2** | **part of a light responsive element** |
| **53** | **H-box**  (CCTACCNNNNNNNCTNNNNA) | **0** | **3** | **0** | **0** | **0** | **0** | **0** | **0** | **0** | **0** | **0** |  |
| **54** | **I-box**  (gGATAAGGTG) | **2,6** | **0** | **2,6** | **2,6** | **2,6** | **5** | **2** | **0** | **2,5** | **2,5** | **1,2,6** | **part of a light responsive element** |
| **55** | **LTR**  (CCGAAA) | **3,6** | **2** | **1,3,6** | **1,6** | **1,4,6** | **0** | **1,3,4,6** | **1,6** | **1,3,4,6** | **1,3,6** | **1,3,6** | **cis-acting element involved in low-temperature responsiveness** |
| **56** | **LS7**  (CAGATTTATTTTT) | **4** | **0** | **0** | **0** | **0** | **0** | **0** | **0** | **0** | **4** | **4** | **Apart of a light responsive element** |

| **S.no** | **Cis elements** | **Oba** | **Obr** | **Ogla** | **Oglu** | **Ol** | **Om** | **On** | **Op** | **Or** | **Osi** | **Osj** | **Function** |
| --- | --- | --- | --- | --- | --- | --- | --- | --- | --- | --- | --- | --- | --- |
| **57** | **MYB**  (CAACAG) | **1,2,3,4** | **1,2,5** | **1,3,4,6** | **1,2,3,4,5,6** | **2,3,4,6** | **2,3,4,5,6** | **1,3,4,6** | **1,4,6** | **1,2,3,4,6** | **1,2,3,4,6** | **1,2,3,4,5,6** |  |
| **58** | **Myb-binding site**  (CAACAG) | **1,2** | **5** | **1,2** | **1,2,5,6** | **2,3** | **5,6** | **1,2,** | **1,6** | **1,2,5,6** | **1,2,** | **1,2,3,5** |  |
| **59** | **MBS**  (CAACTG) | **2** | **1,5** | **2** | **2,3** | **2** | **1,5** | **2** | **3,6** | **2,5** | **2** | **2,3,5,6** | **MYB binding site involved in drought-inducibility** |
| **60**  **Table. 25 Comparison of regulatory elements in of of NLP promoter regions in *Oryza* spp (Contd..)** | **MYC**  (CATTTG) | **2,3,4,5,6** | **2,3,5** | **2,3,4,5,6** | **1,2,4,5,6** | **2,4,5,6** | **1,3,4,5,6** | **3,4,5,6** | **3,4,5,6** | **2,4,5,6** | **2,3,4,5,6** | **2,3,4,5,6** |  |
| **61** | **Myb**  (CAACTG) | **2,3** | **1,2,5** | **2,3** | **2,3,4,6** | **2,3,6** | **1,5,6** | **2,3,6** | **3,6** | **2,3,5,6** | **2,3** | **2,3,5,6** |  |
| **62** | **MYB-like sequence**  (TAACCA) | **3,4** | **1,2** | **3,4** | **3,4,6** | **3,4,6** | **2,4,5,6** | **3,4** | **3,4** | **3,4,6** | **3,4** | **3,4** |  |
| **63** | **MYB recognition site**  (CCGTTG) | **4,5,6** | **0** | **5,6** | **4,5,6** | **4,5** | **5** | **4,5,6** | **4** | **4,5,6** | **4,6** | **6** |  |
| **64** | **MRE**  (AACCTAA) | **0** | **2** | **0** | **0** | **0** | **1** | **0** | **0** | **0** | **6** | **0** | **MYB binding site involved in light responsiveness** |
| **65** | **Myc**  (TCTCTTA) | **0** | **5** | **0** | **0** | **6** | **3,5** | **0** | **0** | **0** | **0** | **3** |  |
| **66** | **MSA-like**  ((T/C)C(T/C)AACGG(T/C)(T/C)A) | **0** | **0** | **0** | **0** | **0** | **0** | **0** | **0** | **0** | **3,6** | **3** | **cis-acting element involved in cell cycle regulation** |
| **67** | **NON**  (CAACGGCCACG) | **0** | **0** | **0** | **0** | **0** | **0** | **0** | **1** | **0** | **0** | **0** |  |
| **68** | **O2-site**  (GATGA(C/T)(A/G)TG(A/G)) | **1,2,6** | **2** | **1,2,6** | **1,2,6** | **0** | **3** | **2,6** | **1** | **2,6** | **2** | **2,3,6** | **cis-acting regulatory element involved in zein metabolism regulation** |
| **69** | **P-box**  (CCTTTTG) | **2** | **0** | **2** | **2,6** | **2,3** | **5,6** | **2,6** | **3,4,6** | **2,6** | **2,** | **2,3** | **gibberellin-responsive element** |
| **70** | **plant_AP-2-like**  (CGACCAGG) | **0** | **0** | **0** | **0** | **0** | **0** | **0** | **0** | **0** | **0** | **2** |  |
| **71** | **RY-element**  (CATGCATG) | **0** | **5** | **0** | **0** | **0** | **0** | **0** | **0** | **0** | **0** | **0** | **cis-acting regulatory element involved in seed-specific regulation** |
| **72** | **STRE**  (AGGGG) | **1,2,3,4,5,6** | **1,2,3,4** | **1,2,3,4,5,6** | **1,2,4,5,6** | **1,2,3,4,5,6** | **1,4,5** | **1,2,4,5,6** | **1,3,4,5,6** | **1,2,3,4,5,6** | **1,2,3,4,5,6** | **1,2,3,4,5,6** |  |

| **S.no** | **Cis elements** | **Oba** | **Obr** | **Ogla** | **Oglu** | **Ol** | **Om** | **On** | **Op** | **Or** | **Osi** | **Osj** | **Function** |
| --- | --- | --- | --- | --- | --- | --- | --- | --- | --- | --- | --- | --- | --- |
| **73** | **Sp1**  (GGGCGG) | **3,4** | **1,3** | **3,4** | **4** | **4** | **1,3,4** | **4,** | **3,4,5** | **3,4,5** | **3,6** | **1,3,4** | **light responsive element** |
| **74** | **sbp-CMA1c**  (CTTTATCTCTTCC) | **0** | **0** | **0** | **0** | **0** | **5** | **0** | **0** | **0** | **0** | **0** | **Apart of a light responsive element** |
| **75** | **TATA**  (TATAAAAT) | **1,5** | **2** | **0** | **0** | **3** | **3** | **0** | **0** | **4** | **0** | **5** |  |
| **76** | **TATA-box**  (ATATAT) | **1,3,4,6** | **2,4,5** | **1,4,5,6** | **1,3,4,5,6** | **4,5,6** | **1,3,4,5,6** | **1,3,4,5,6** | **3,4,5,6** | **1,3,4,5,6** | **1,4,5,6** | **1,2,3,4,5,6** | **core promoter element around -30 of transcription start** |
| **77** | **TCT-motif**  (TCTTAC) | **1,3** | **4** | **3,4** | **3** | **6** | **3** | **3,4** | **3,4,5,6** | **3** | **3,5,6** | **3,4,5,6** | **part of a light responsive element** |
| **78** | **TGACG-motif**  (TGACG) | **2,3,5,6** | **1,3** | **2,5,6** | **2,3,5,6** | **2,3,5,6** | **2,5** | **2,5,6** | **1,5,6** | **2,5,6** | **2,5,6** | **1,2,5,6** | **cis-acting regulatory element involved in the MeJA-responsiveness** |
| **79** | **TCA**  (TCATCTTCAT) | **3,6** | **0** | **6** | **3,6** | **0** | **3** | **3,6** | **0** | **3,6** | **0** | **3** |  |
| **80** | **TCA-element**  (CCATCTTTTT) | **4** | **0** | **4** | **4** | **3** | **3,5** | **0** | **3,4** | **0** | **4** | **4** | **cis-acting element involved in salicylic acid responsiveness** |
| **81** | **TGA-element**  (AACGAC) | **4,6** | **2** | **4,6** | **4,6** | **3,4** | **1,3,4,** | **3,6** | **0** | **3,6** | **0** | **2,3,4** | **auxin-responsive element** |
| **82** | **TCCC-motif**  (TCTCCCT) | **5** | **2** | **5** | **5,6** | **3,5** | **6** | **5,6** | **5,6** | **6** | **5** | **3,4,5** | **part of a light responsive element** |
| **83** | **TATC-box**  (TATCCCA) | **0** | **5** | **0** | **0** | **0** | **0** | **0** | **0** | **0** | **0** | **0** | **cis-acting element involved in gibberellin-responsiveness** |
| **84** | **TC-rich repeats**  (GTTTTCTTAC) | **0** | **5** | **0** | **0** | **0** | **2,3** | **0** | **4,** | **0** | **0** | **0** | **cis-acting element involved in defense and stress responsiveness** |
| **85** | **Unnamed__1**  (CGTGG) | **1,2,3,5,6** | **1,3** | **1,2,3,5,6** | **1,2,3,5,6** | **1,2,3,5** | **1,5,6** | **1,2,3,5,6** | **1,3,4,5** | **1,2,3,5,6** | **1,2,3,6** | **1,2,3,5,6** |  |
| **86** | **Unnamed__4**  (CTCC) | **1,2,3,4,5,6** | **1,2,3,4,5** | **1,2,3,4,5,6** | **1,2,3,4,5,6** | **1,2,3,4,5,6** | **1,2,3,4,5,6** | **1,2,3,4,5,6** | **1,3,4,5,6** | **1,2,3,4,5,6** | **1,2,3,4,5,6** | **1,2,3,4,5,6** |  |

| **S.no** | **Cis elements** | **Oba** | **Obr** | **Ogla** | **Oglu** | **Ol** | **Om** | **On** | **Op** | **Or** | **Osi** | **Osj** | **Function** |
| --- | --- | --- | --- | --- | --- | --- | --- | --- | --- | --- | --- | --- | --- |
| **85** | **Unnamed__1**  (CGTGG) | **1,2,3,5,6** | **1,3** | **1,2,3,5,6** | **1,2,3,5,6** | **1,2,3,5** | **1,5,6** | **1,2,3,5,6** | **1,3,4,5** | **1,2,3,5,6** | **1,2,3,6** | **1,2,3,5,6** |  |
| **86** | **Unnamed__4**  (CTCC) | **1,2,3,4,5,6** | **1,2,3,4,5** | **1,2,3,4,5,6** | **1,2,3,4,5,6** | **1,2,3,4,5,6** | **1,2,3,4,5,6** | **1,2,3,4,5,6** | **1,3,4,5,6** | **1,2,3,4,5,6** | **1,2,3,4,5,6** | **1,2,3,4,5,6** |  |
|  |  |  |  |  |  |  |  |  |  |  |  |  |  |
| **87** | **Unnamed__2**  (CCCCGG) | **6** | **3** | **4,6** | **6** | **1,6** | **6** | **6** | **0** | **6** | **4,6** | **1,2,4,6** |  |
| **88** | **Unnamed__16**  (GCTGCCCGTC) | **0** | **0** | **0** | **0** | **1,3** | **0** | **0** | **0** | **0** | **0** | **2,5** |  |
| **89** | **Unnamed__10**  (TCCACGTAGA) | **0** | **0** | **0** | **0** | **0** | **0** | **0** | **3** | **0** | **0** | **0** |  |
| **90** | **Unnamed__12**  (TCCACGTAGA) | **0** | **0** | **0** | **0** | **0** | **0** | **0** | **3** | **0** | **0** | **0** |  |
| **91** | **Unnamed__14**  (TCCACGTAGA) | **0** | **0** | **0** | **0** | **0** | **0** | **0** | **3** | **0** | **0** | **0** |  |
| **92** | **Unnamed__8**  (TCCACGTAGA) | **0** | **0** | **0** | **0** | **0** | **0** | **0** | **3** | **0** | **0** | **0** |  |
| **93** | **W box**  (TTGACC) | **1,2** | **5** | **1,2,6** | **1,2,6** | **3,6** | **1,6** | **1,2,6** | **1,5,6** | **1,2,6** | **1,2,3** | **1,2,3,6** |  |
| **94** | **WRE3**  (CCACCT) | **1,2,3,6** | **1,3,5** | **1,2,5,6** | **1,2,6** | **2,6** | **1,6** | **1,2,3,6** | **1,6** | **1,2,3,6** | **1,5** | **1,2,3,5,6** |  |
| **95** | **WUN-motif**  (AAATTACTA) | **0** | **0** | **0** | **5** | **0** | **1** | **0** | **5** | **0** | **0** | **0** |  |

**Supplementary Fig 1 Hydroponic evaluation of rice genotypes. High nitrate: Low ammonia ratio (T1: 6.5mM Nitrate: 1mM Ammonium), Low nitrate: High ammonia (T2: 6.5mM Ammonium: 1mM Nitrate), Low N (T3: 0.24mM Ammonium Nitrate)**

**
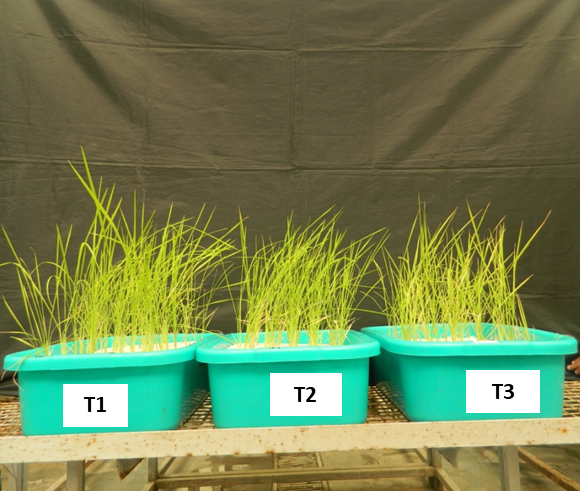
**

**Supplementary Fig 2 Comparison of growth of rice genotypes Apo, IR-83929-B-B-291-3-1-1 (IR-3-1-1), and Nerica L-42 (NL-42), and Pusa Basmati 1 (PB1) in hydroponics and receiving different nitrogen treatments; T1: 6.5mM Nitrate: 1mM Ammonium, T2: 6.5mM Ammonium: 1mM Nitrate, T3: 0.24mM Ammonium Nitrate.**


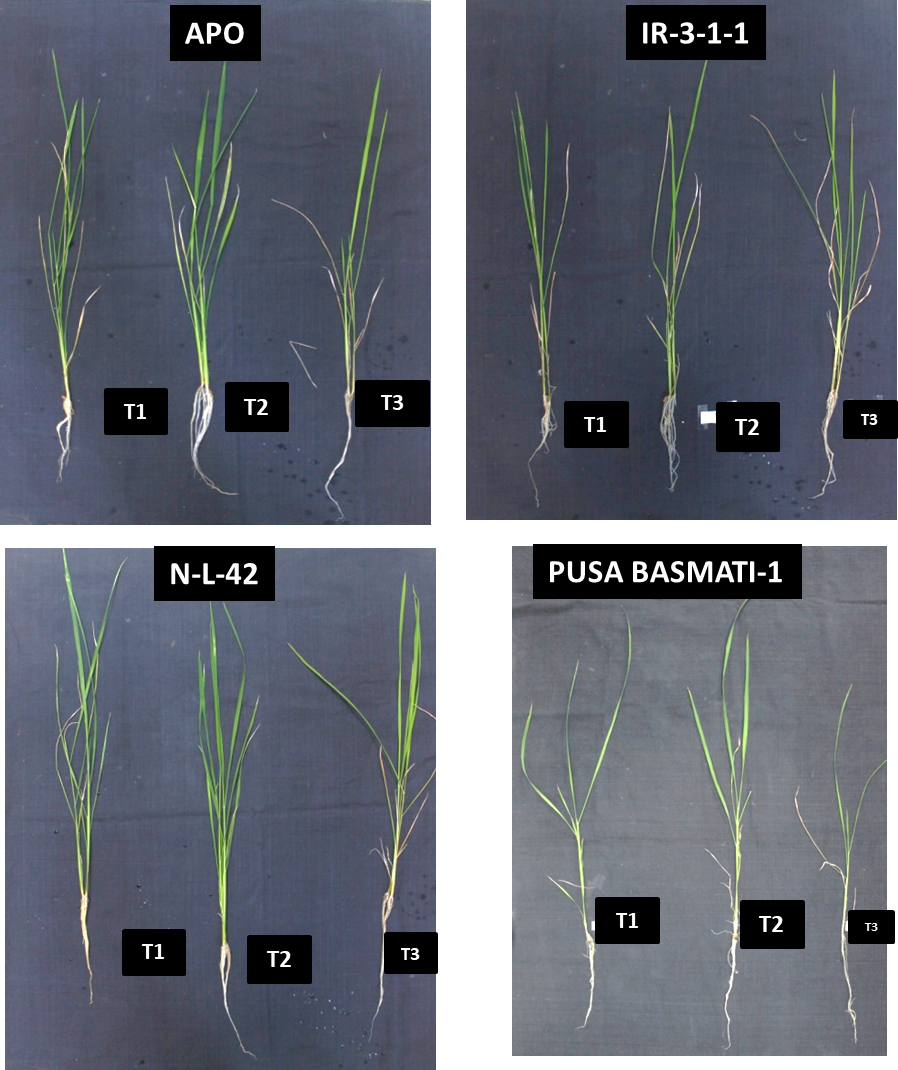


**Supplementary Fig 3 (a) Gene structure of of *NLP* homologues in *Oryza sativa* Japonica (b) Chromasomal localization of *NLP* homologues in *Oryza sativa* Japonica**


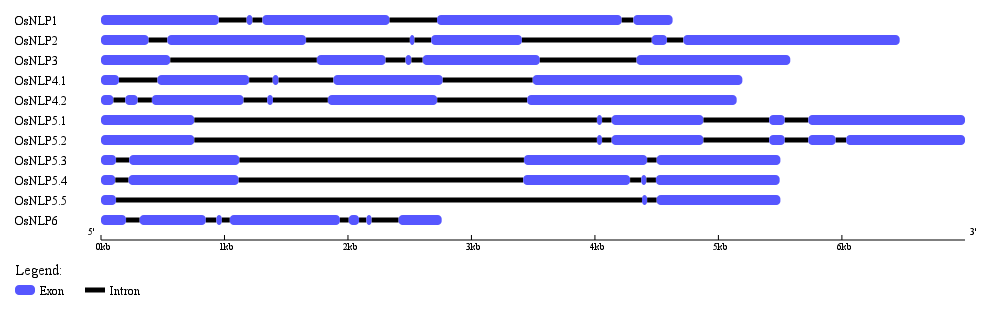


**(a)**

**(b)**

**
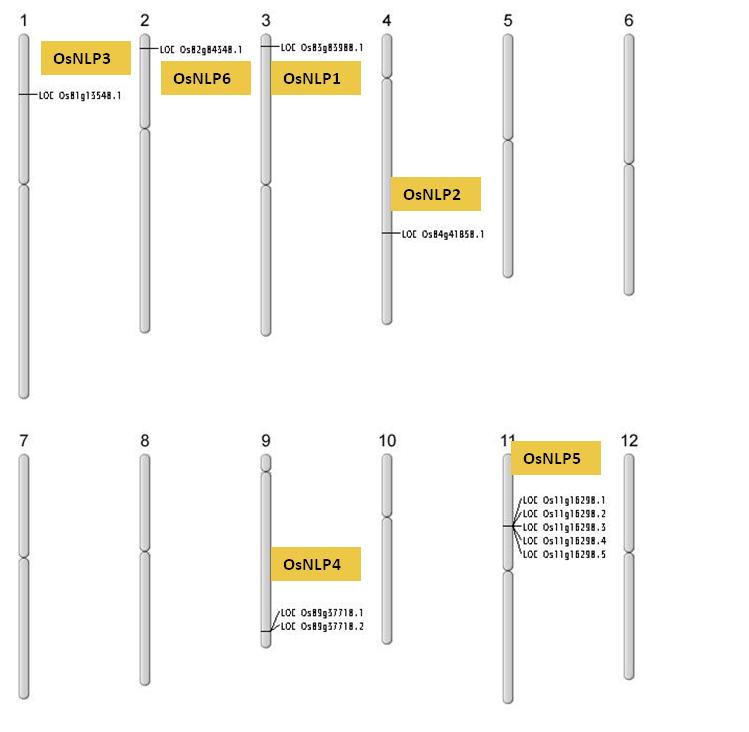
**

**Supplementary Fig 4 Phylogenetic analysis of the *NLP* genes in *Arabidopsis thaliana* and 11 *Oryza spp***


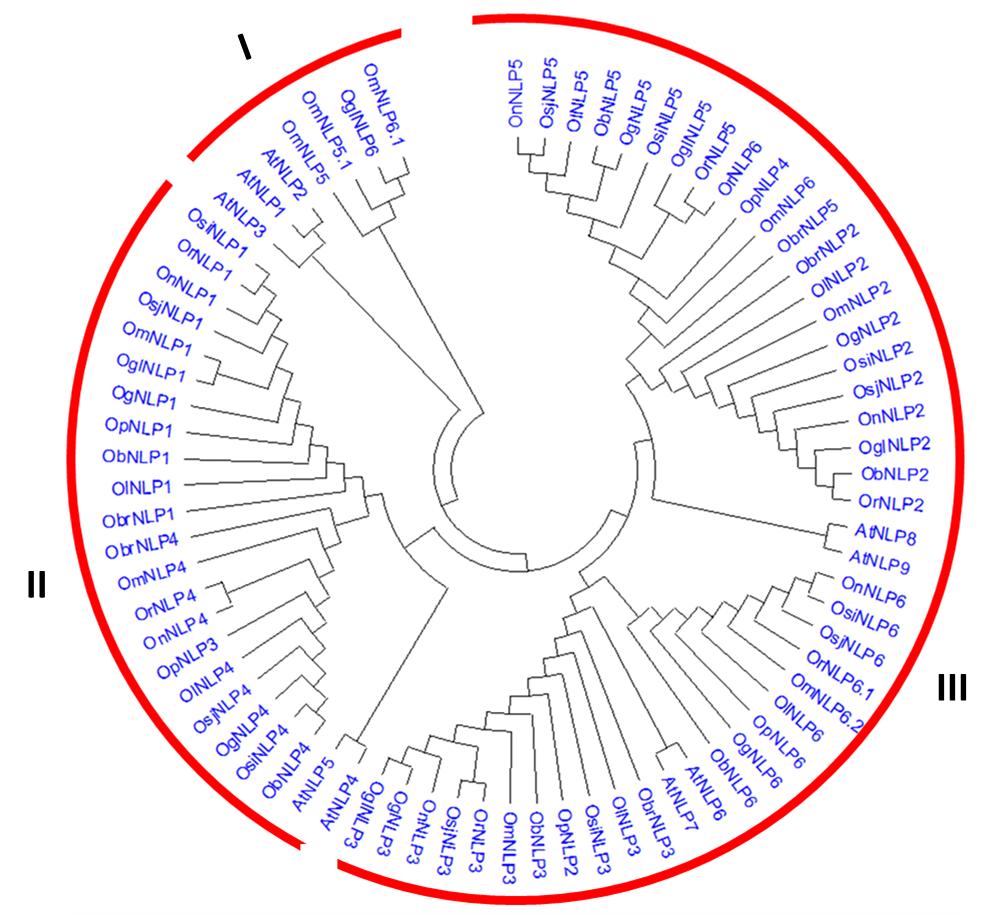


**
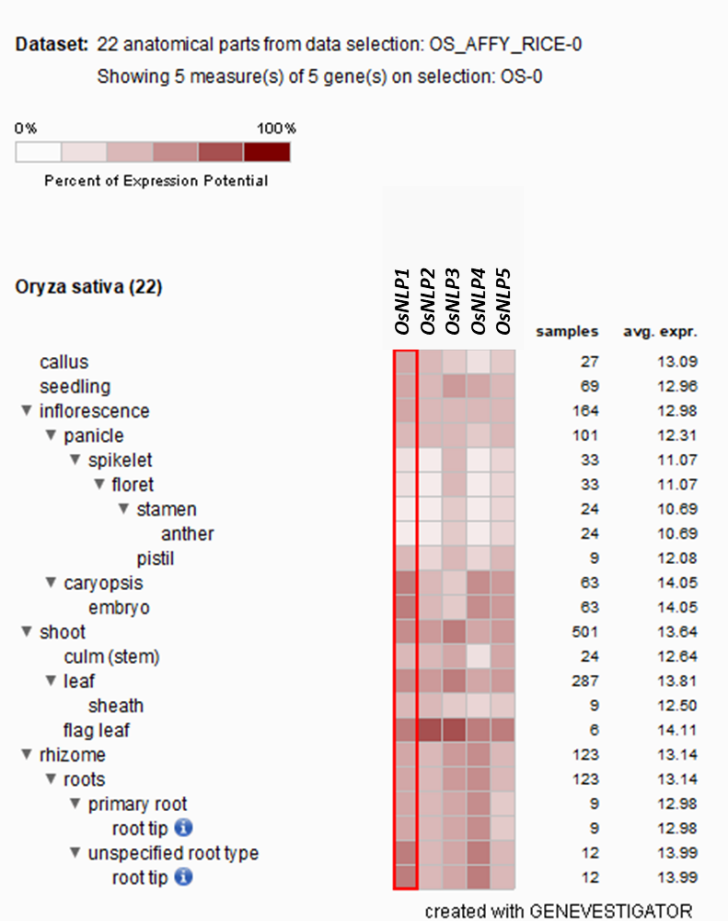

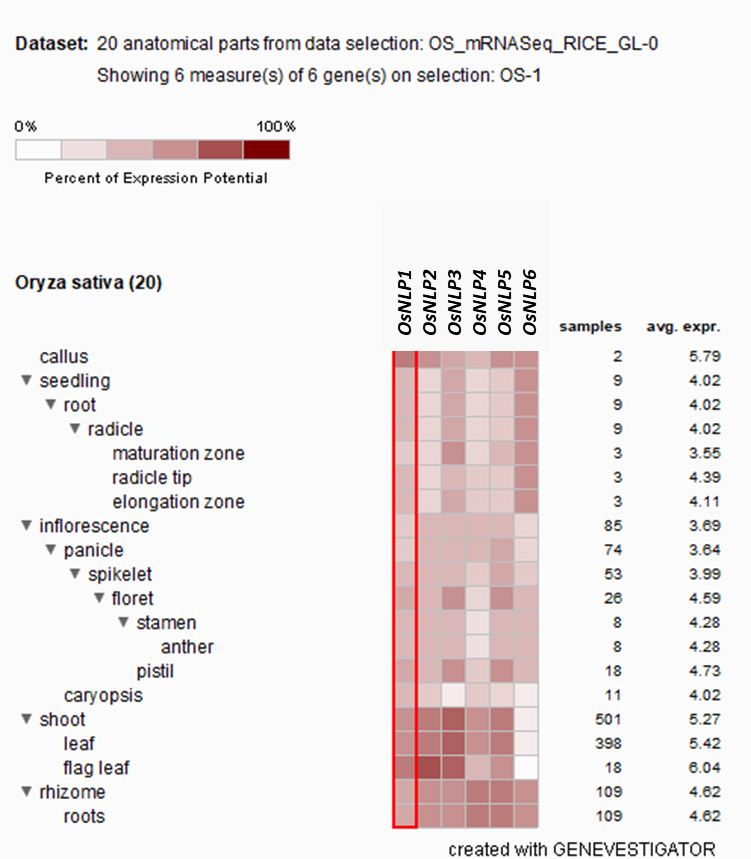
**
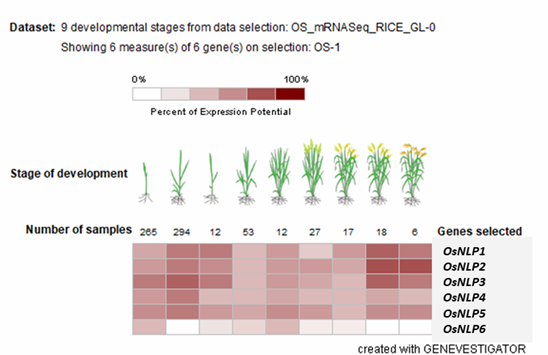
**
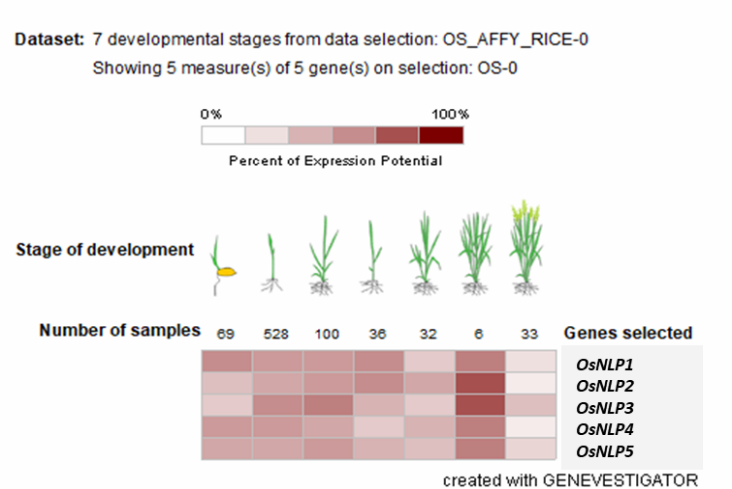
Supplementary Fig 5 Expression analyses of *NLP* genes at different developmental stages and anatomical parts using Genevestigator database. (a, b) Expression profiling based on microarray data (c, d) Expression profiling using mRNAseq data**

**(d)**

**(c)**

**(b)**

**(a)**

**Supplementary Fig 6 Expression analyses of NLP genes hormones (a) and other perturbations showing significant expression changes at P value ≤ 0.05 (b) using Genevestigator database.**


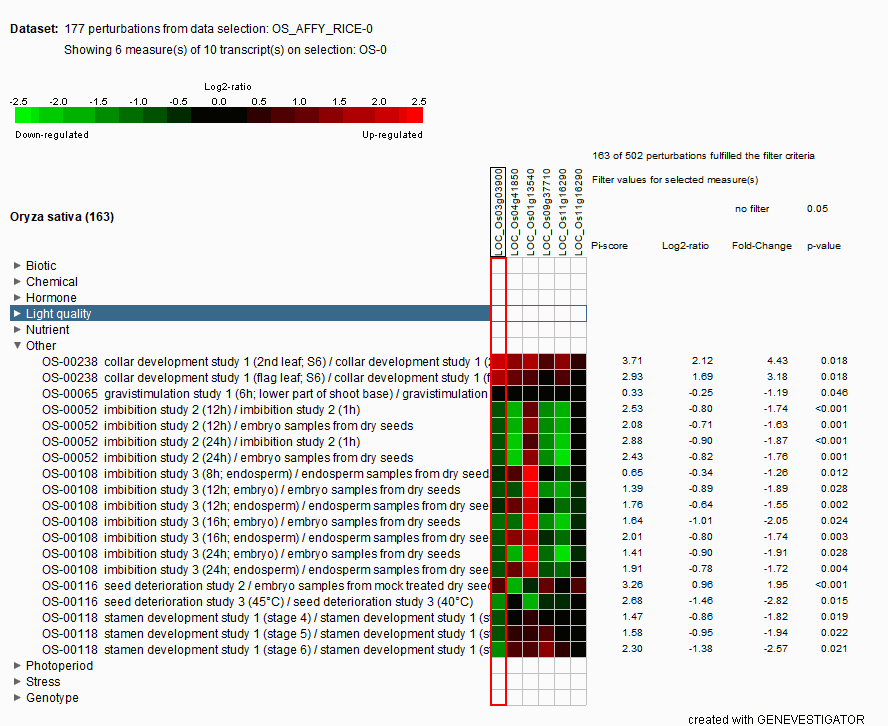

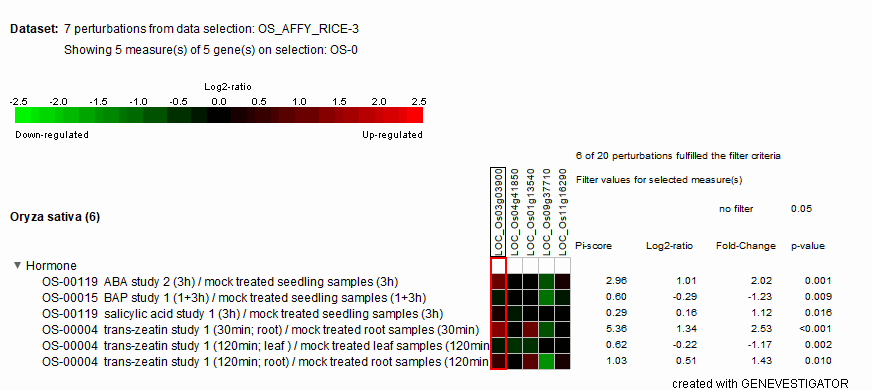


**Supplementary Fig 7 Expression analysis of *NLP* genes in response to nutrient (a) and abiotic stress (b) perturbations showing significant expression changes at P value ≤0.05 (b) using Genevestigator database.**

**(a)**


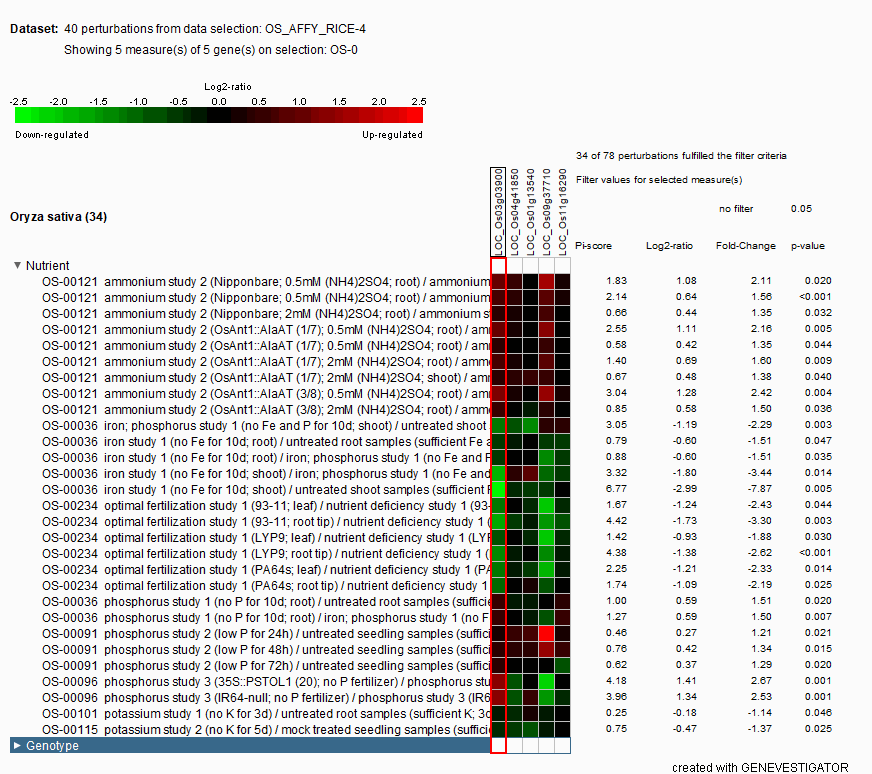


**(b)**


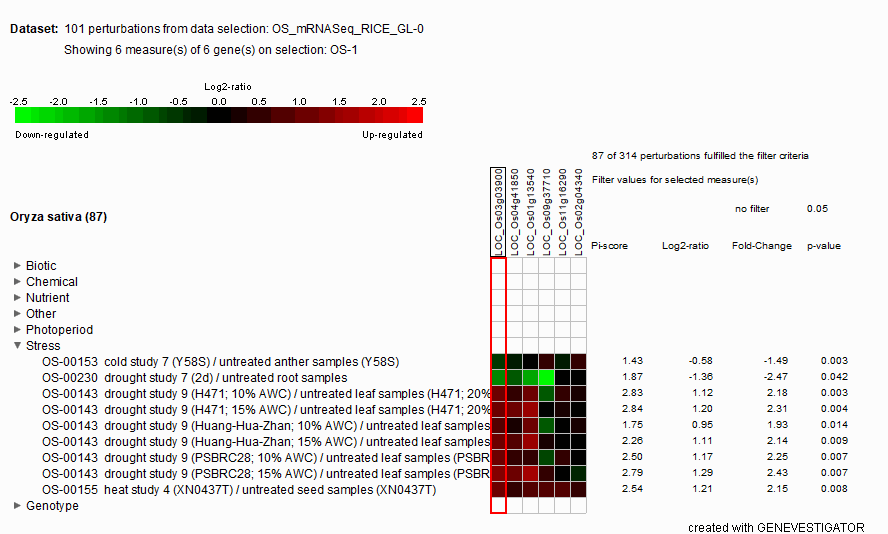


**Supplementary Fig 8: Protein-protein interaction network of NLP proteins from rice. Networks pertaining to *Oryza sativa* Japonica NLPs are depicted in the figure using SMART (**[**http://smart.embl-heidelberg.de/**](http://smart.embl-heidelberg.de/) **) followed by prediction of interaction partners and networks using STRING tool (**[**http://string-db.org/**](http://string-db.org/)**).**
